# Characterization of the mitochondrial genome of an ancient amphipod *Halice* sp. MT-2017 (Pardaliscidae) from 10,908 m in the Mariana Trench

**DOI:** 10.1038/s41598-019-38735-z

**Published:** 2019-02-22

**Authors:** Jun-yuan Li, Cong Zeng, Guo-yong Yan, Li-sheng He

**Affiliations:** 1 Institute of Deep-sea Science and Engineering, Chinese Academy of Sciences, Sanya, Hainan China; 2 University of Chinese Academy of Sciences, Beijing, China; 3 Hunan Agricultural University, Changsha, Hunan China

## Abstract

Small amphipods (*Halice* sp. MT-2017) with body length <1 cm were collected from the Challenger Deep (~10,920 m below sea level). The divergence time of their lineage was approximately 109 Mya, making this group ancient compared to others under study. The mitochondrial genome of *Halice* sp. shared the usual gene components of metazoans, comprising 13 protein coding genes (PCGs), 22 transfer RNAs (tRNAs), and 2 ribosomal RNAs (rRNAs). The arrangement of these genes, however, differed greatly from that of other amphipods. Of the 15 genes that were rearranged with respect to the pancrustacean gene pattern, 12 genes (2 PCGs, 2 rRNAs, and 8 tRNAs) were both translocated and strand-reversed. In contrast, the mitochondrial genomes in other amphipods never show so many reordered genes, and in most instances, only tRNAs were involved in strand-reversion-coupled translocation. Other characteristics, including reversed strand nucleotide composition bias, relatively higher composition of non-polar amino acids, and lower evolutionary rate, were also identified. Interestingly, the latter two features were shared with another hadal amphipod, *Hirondellea gigas*, suggesting their possible associations with the adaptation to deep-sea extreme habitats. Overall, our data provided a useful resource for future studies on the evolutionary and adaptive mechanisms of hadal faunas.

## Introduction

Amphipods are widely spread in both marine and freshwater habitats^1^. Even in some extreme environments, such as the Arctic ice pack^2^, Antarctic tide pools^3^, and subterranean waters^4^, amphipods are important constituents of macrofaunal communities. The hadal zone, which is the deepest part of the ocean (from approximately 6,000 m to 11,000 m), is a harsh environment characterized by high hydrostatic pressure, food supply scarcity, constant darkness, and low temperature^5^. Amphipods have emerged as a representative fauna in this ecosystem, particularly at depths exceeding 8,000 m^6^. Because of their ease of capture by baited trapping^7–9^, these animals represent one of the few faunas that can be readily obtained in large numbers and diversities. The studies on them would provide statistically meaningful data for various aspects, including feeding habits^10^, population genetics^11,12^, and even adaptive mechanisms in deep-sea extreme environments^13^.

Pardaliscidae is one of the families in Amphipoda attractive to the researchers of deep sea faunas as most representatives of them inhabit in the abyssal or hadal environment^14^, and some members even extend to the greatest depth below 10,000 m^15^. During our sampling campaign to the Mariana Trench in March 2017, individuals of amphipods in Pardaliscidae (*Halice* sp. MT-2017) were obtained at several sampling points (~11,000 m deep). They showed a body length less than 1 cm and had a similar density as another hadal amphipod, *Hirondellea gigas*, which was a common species in Mariana Trench and could be collected below 10,000 m as well.

The mitochondrial genome has been established as a useful tool for studying phylogeny, molecular evolution, and phylogeography^16–19^ because of its conserved gene content, easily accessible nature, and relatively high evolutionary rate^17,20,21^. Currently, sequences of mitochondrial genomes from amphipods within seven superfamilies, Allocrangonyctoidea, Calliopioidea, Caprelloidea, Gammaroidea, Hadzioidea, Lysianassoidea, and Talitroidea, have been reported. Comparative studies across these seven superfamilies indicated that most amphipod mitochondrial genomes shared typical metazoan gene content comprising 37 genes—13 protein coding genes (PCGs), 2 ribosomal genes (rRNAs), and 22 transfer genes (tRNAs), as well as a putative non-coding control region (CR)^22–24^. However, the order of these genes differed across superfamilies^24^, suggesting that there was an extensive gene rearrangement process during the evolution of Amphipoda. Moreover, characteristics of certain superfamilies have been identified. For example, the mitochondrial DNAs of Metacrangonyctidae (Hadzioidea) showed an opposite strand nucleotide bias to that observed in the majority of crustacean mitochondrial genomes, but this reversal of strand bias has seldom been seen in other amphipod superfamilies^24^.

Among all available mitochondrial genomes of amphipods, data from hadal extreme environments are so scarce that only one mitochondrial genome of *H. gigas* (Lysianassoidea) has been reported to date^25^. Characterization of a novel mitochondrial sequence from the hadal trench, therefore, would definitely contribute to the available deep-sea genetic resources. In the present study, the mitochondrial genome of *Halice* sp. MT-2017 (Pardaliscidae) collected from nearly 11,000 m deep in the Mariana Trench was sequenced, annotated, and compared with the mitochondrial genomes of other members from shallower habitats. The features of its gene arrangement, the bias of strand nucleotide composition, and the codon/amino acid usage pattern were also described, furthermore, the evolutionary characteristics of the mitochondrial genomes were discussed.

## Results and Discussion

### Sampling information of *Halice* sp. MT-2017

**Figure 1:**
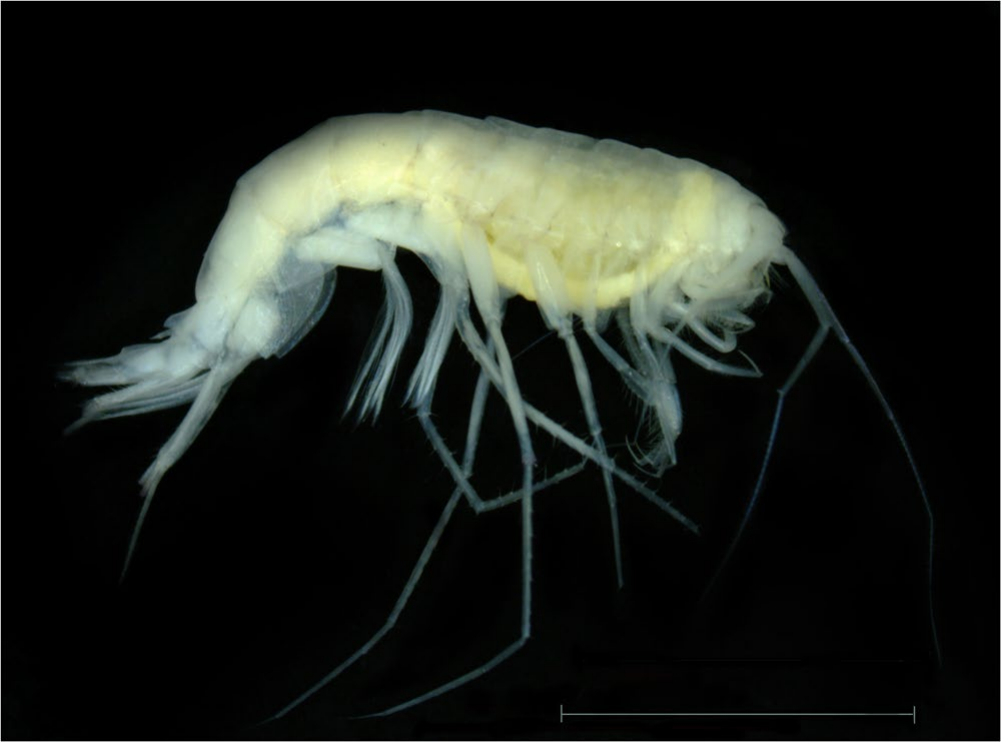
An individual *Halice* sp. MT-2017 collected from 10,908 m in the Mariana Trench. Scale bar: 0.5 cm.

*Halice* sp. MT-2017 (Fig. 1) was collected from the Mariana Trench during the TS-03 cruise in March 2017. Together with *H. gigas*, *Halice* sp. MT-2017 were collected from all eight sampling sites below 10,000 m (Table S1). Statistical analysis indicated no significant difference (*P* > 0.5) between these two hadal species in terms of their abundance at depths below 10,000 m. From 7,000–9,000 m, no *Halice* sp. MT-2017 specimens were trapped; however, many *H. gigas* specimens were collected in that depth range (Table S1). This suggests that *Halice* sp. MT-2017 could be a native species in the hadal environment. Moreover, at the Challenger Deep site, *Halice* sp. is another common species besides *H. gigas*.

### Mitochondrial genome organization

A total of 57,366,782 clean reads (8,605,017,300 base pairs [bp]) were generated by Illumina HiSeq sequencing. After assembling, an approximately complete mitochondrial genome of the hadal *Halice* sp. MT-2017 was obtained with a coverage depth of 400.302×. The total length of the assembled mitochondrial genome was 15,199 bp (GenBank accession ID: MH294484), which was comparable to the mitochondrial DNAs from other amphipods ranging from 14,113 to 18,424 bp^23^. The nearly complete mitochondrial genome contained 37 genes, comprising 13 PCGs, 22 tRNAs, and 2 rRNAs (Table 1), which showed the same components as other metazoan mitochondrial genomes^22^. Because the CR was a constituent part for the metazoan mitochondrial DNAs^26^, it was assumed to be located in the unprocurable region between the two rRNA genes. The ubiquity of the repeated sequences and low complexity in the CR impeded automatic assembly *in silico*. Amplifications by polymerase chain reaction (PCR) also failed to complement the remaining CR, probably because of the AT-abundant regions, poly(A)/poly(T) stretches, or hairpin structures^27^.

**Table 1:** Gene content of the *Halice* sp. MT-2017 mitochondrial genome.

Feature	Strand	From	To	Size (bp)	Start codon	Stop codon	GC content (%)	Intergeic nucleotides
rrnL	L	61	1,121	1,061			19.79	0
trnL1(tag)	L	1,122	1,188	67			32.84	0
nad1	L	1,189	2,109	921	ATA	TAG	26.71	25
trnP(tgg)	L	2,135	2,199	65			13.85	10
nad5	L	2,210	3,931	1,722	ATT	TAG	26.66	−1
trnF(gaa)	L	3,931	3,993	63			20.63	11
trnE(ttc)	H	4,005	4,069	65			15.38	−32
trnV(tac)	L	4,038	4,093	56			16.07	−22
trnN(gtt)	H	4,072	4,136	65			18.46	2
trnS1(tct)	H	4,139	4,193	55			25.45	5
trnA(tgc)	H	4,199	4,260	62			19.35	30
trnQ(ttg)	H	4,291	4,355	65			18.46	8
trnC(gca)	H	4,364	4,429	66			22.73	9
trnY(gta)	H	4,439	4,505	67			16.42	5
trnI(gat)	L	4,511	4,575	65			29.23	0
trnM(cat)	L	4,576	4,639	64			25.00	1
nad2	L	4,641	5,633	993	ATT	TAA	22.96	0
trnW(tca)	L	5,634	5,698	65			16.92	0
cox1	L	5,699	7,240	1,542	ATT	TAA	33.33	−5
trnL2(taa)	L	7,236	7,298	63			25.40	0
cox2	L	7,299	7,971	673	ATA	T(AA)	30.01	0
trnK(ttt)	L	7,972	8,040	69			21.74	−5
trnD(gtc)	L	8,036	8,099	64			20.31	0
atp8	L	8,100	8,258	159	ATC	TAA	25.79	−7
atp6	L	8,252	8,920	699	ATG	TAA	28.55	2
cox3	L	8,923	9,711	789	ATG	TAA	32.07	−1
trnG(tcc)	L	9,711	9,771	61			18.03	0
nad3	L	9,772	10,125	354	ATT	TAA	28.81	0
trnR(tcg)	L	10,126	10,189	64			18.75	7
trnH(gtg)	H	10,197	10,261	65			12.31	3
nad4	H	10,265	11,572	1,308	ATG	TAA	29.05	−7
nad4L	H	11,566	11,856	291	ATG	TAA	24.74	27
trnT(tgt)	L	11,884	11,946	63			11.11	−3
nad6	L	11,944	12,444	501	ATT	TAA	25.15	2
cytb	L	12,447	13,562	1,116	ATG	TAA	30.65	9
trnS2(tga)	L	13,572	13,633	62			11.29	100
rrnS	L	13,734	14,468	735			22.59	0
control region	H*	14,469	15,199	731			5.61	0

Note: *The control region of the mitochondrial genome was hypothetically located on the H strand inferred from the reversals of strand asymmetry in protein coding genes.

### Phylogenetic analysis and divergence time estimation

The hadal *Halice* sp. MT-2017 from the Mariana Trench clustered robustly with the abyssal species collected at 2567.6 m from the Iceland Basin^28^, and separated from others in shallower water (289.4–510.9 m) using Bayesian and maximum likelihood methods based on partial cytochrome c oxidase subunit I (*cox1*) barcodes (Fig. S1). In terms of all the taxa in *Halice* used in the phylogram, although none of them had been nominated a specific name, the genetic distances between them, calculated by the *p*-distance, were large enough to delineate them at the species level (Table S2), considering that 0.03 of the *p*-distance had commonly been used as a threshold for amphipod species demarcation^28^. The vertical and geographic distribution of *Halice* discovered in hadal trenches and Icelandic waters conformed to the “tropical submergence” hypothesis, which proposed that closely related species lived in shallower waters at high latitudes and deeper waters at low latitudes, a reflection of adaptation to cold temperature^29^. Other evidence can also be seen in hydrothermal vents, an ecosystem different from those of Icelandic waters and hadal trenches, where *Halice* (*H. hesmonectes*, with no barcodes submitted) were distributed only in the vicinity of low temperature vent openings (2–8 °C)^30^. Considering the fact that *Hirondellea* (which *H. gigas* belonged to) was a shallower-Antarctic and deep-sea genus^31^, also in line with “tropical submergence”, hadal amphipods supposedly originated or derived from relatively higher latitudes.

Interfamilial phylogenetic analyses were performed with a dataset of 13 amphipods as ingroup based on the concatenated nucleic acid and amino acid sequences of 13 protein coding genes (PCGs) using the maximum likelihood (ML) method. The topologies of the two phylogenetic trees were nearly congruent in our study, illustrating eight separate clades which corresponded to eight identified families or superfamilies (Fig. S2). The *p*-distance analysis showed that irrespective of the clade represented by Pardaliscidae, the inter-group genetic distances in the other seven superfamilies ranged from 32.43% (between Lysianassoidea and Talitroidea) to 40.28% (between Hadzioidea and Caprelloidea) (Table S3). Regarding Pardaliscidae, the genetic distance to the superfamily Talitroidea was the shortest (38.65%) and the distance to Caprelloidea was the longest (41.52%). Given the minimal inter-clade divergence for Pardaliscidae (38.65%) falling into the range of the inter-superfamily divergence defined by the other seven clades (32.43–40.28%), it is reasonable to discriminate Pardaliscidae from the other groups at a superfamily level.

**Figure 2:**
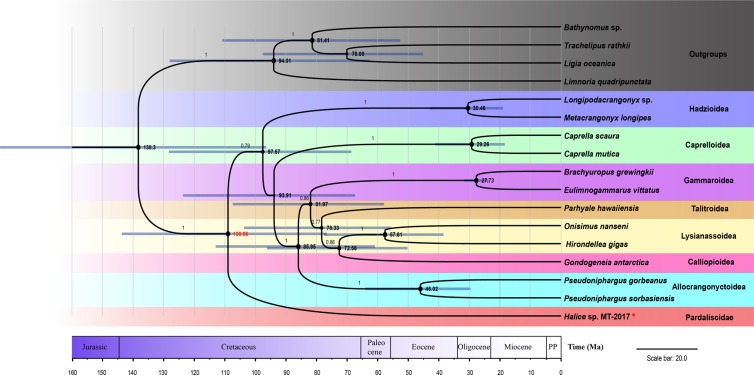
Chronogram of eight superfamilies in Amphipoda inferred using the Bayesian relaxed-molecular clock method. *denoted *Halice* sp. MT-2017 specimens collected from the hadal trench in the present study. The resulting superfamilies were shown adjacent to the branches. Four sequences from Isopoda were used as outgroups. The size of the nodes in the tree corresponded to the clade credibility. Node bars indicated 95% credible intervals of the estimated divergence time. The italic number indicated the placement for the calibration taxa. The accession numbers of the sequences used in the phylogenetic analysis were listed in Supplementary Table S8.

The dendrogram with 95% credible intervals (CIs) for divergence time estimation was constructed from Bayesian analysis, revealing a similar topology to the ML trees with high posterior probabilities (Fig. 2). Although the credible intervals were large in some nodes due to the lack of fossil calibration, some interesting results still could be inferred. The hadal amphipods under study (*Halice* sp. MT-2017 and *H. gigas*) were polyphyletic, with members diverging at different times and belonging to different taxonomic classifications. The hadal cladogenesis for Pardaliscidae (which *Halice* belongs to) was approximately 109 Mya (95% CI: 76.58–143.57 Mya) during the Cretaceous period. They could belong to one of the ancient faunas that survived the catastrophe in the Late Cretaceous (Maestrichtian)^32,33^, during which uncertain factors, such as comet impacts, volcanic eruptions, acid rain, sea level transgressions, and sea level regressions, eradicated more than half of the marine species^33^. The discovery of relatively primitive relict groups in the deep sea has also occurred for many other taxa, such as starfishes, isopods, and bivalves. The great depths of the ocean are speculated to provide refuge for their continued existence^34^. Nevertheless, in more cases, the deep-sea faunas were relatively “young,” occurring no earlier than the Cenozoic period (65.5 Mya to present)^15^. *H. gigas* in the present study dated back to around 58 Mya (95% CI: 38.46–77.50 Mya), a divergence time earlier than that of the *Halice* lineage, and possibly went through the boundary of the Paleocene and Eocene periods, during which a climate-driven anoxia or dysoxyia caused extinctions in the deep sea^35,36^. Only the taxa with strong resistance would escape the radical extinction and show allopatric speciation^36,37^. Considering the significant discrepancy in body sizes (<1 cm for *Halice* sp. MT-2017 vs 2–5 cm for *H. gigas*, trapped concurrently), and the phylogenetic status of *Halice* sp. MT-2017 and *H. gigas*, the evolution of these two species could have proceeded by different biological processes and physiological mechanisms.

### Mitochondrial gene rearrangement

**Figure 3:**
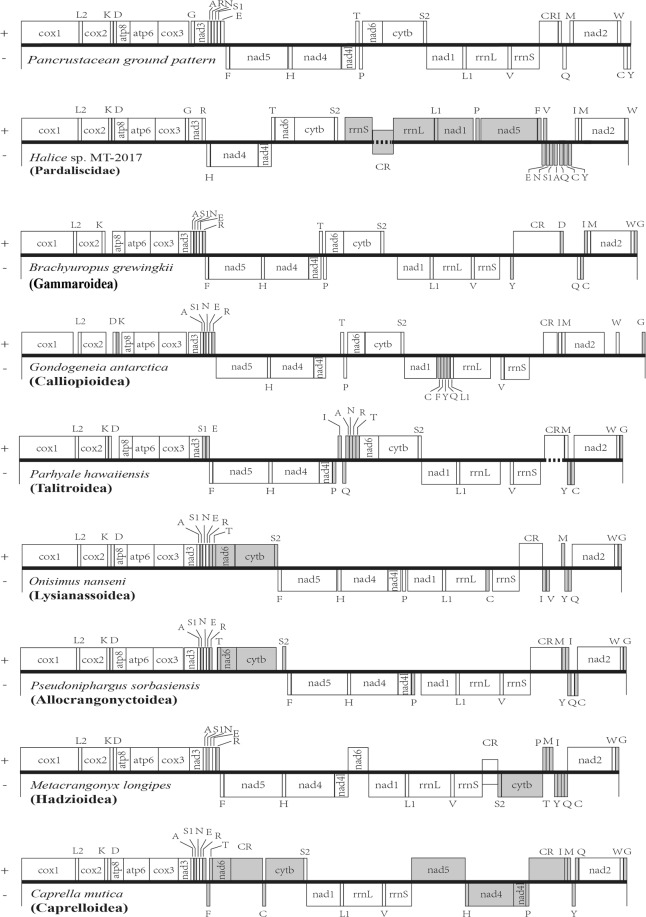
Comparison of gene arrangements among different superfamilies in Amphipoda. Genes with rearranged locations related to the hypothetical pancrustacean gene pattern were highlighted in gray color. The genes above the line were encoded by the light (or plus) strand, whereas, the ones below the line were encoded by the heavy (or minus) strand. The CRs of *Halice* sp. MT-2017 and Metacrangonyctidae were in the middle of the line to denote the uncertainty of the replication origin. Segments with no explicit sequences were shown by dotted lines in some of the central bold lines because of the technical barriers. Transfer RNAs were labelled as their corresponding single letter amino acid code apart from L1, L2, S1, and S2 for trnL (CUN), trnL (UUR), trnS (AGN), and trnS (UCN), respectively. The name of the superfamily was in bold and bracketed. The representative of the superfamily was listed below the central line. The mitochondrial genome of *H. gigas* was excluded from comparison because of its uncertain gene order caused by the unjoined contigs.

The gene arrangements of eight superfamilies were compared to the hypothetical ancestral pancrustacean (hexapods and crustaceans) gene order^38^ (Fig. 3). There were 15 genes in *Halice* sp. MT-2017 that showed altered locations, among which 12 were both translocated and strand-reversed. The reverse-stranded-translocation event, which has rarely been discovered in the rRNAs and PCGs of other amphipod mitochondrial genomes, resulted in the switch of transcriptional polarity in relevant genes of *Halice* sp. MT-2017, including two rRNAs, two PCGs (*nad1*, *nad5*), and eight tRNA genes (*trnL* [CUN], *trnP*, *trnF*, *trnE*, *trnV*, *trnN*, *trnS* [AGY], and *trnA*). Regarding PCGs, gene rearrangements in other amphipod mitochondrial genomes mostly occurred in *nad6* and *cytb*^27^. For *Halice* sp. MT-2017, however, the gene orders of *nad6* and *cytb* were identical to those of the pancrustacean ground pattern. Alternatively, the changes in the order of PCGs were focused on the rearrangements of *nad1* and *nad5* (Fig. 3). This pattern of change for PCGs was not exclusive to *Halice* sp. MT-2017, but has only been seen in some specific cases, such as the rearrangements of *nad1* in *Pallaseopsis kesslerii* (Gammaroidea)^23^ and *nad5* in *Caprella mutica* (Caprelloidea)^39^. Regarding the altered gene order of tRNAs with respect to the ancestral pancrustacean pattern, the translocation of *trnG* and the typical derived pattern of the gene string *trnA*, *trnS* (AGN), *trnN*, *trnE*, and *trnR* were assumed to be two apomorphic features in the extant amphipod species^39,40^ (Fig. 3). However, *Halice* sp. MT-2017 retained the unaltered position of *trnG* relative to the location in the ancestral pancrustacean mitochondrial genome and the alternation of the tRNA order gave rise to a unique tRNA string (*trnE*, *trnN*, *trnS* [AGY], *trnA*, *trnQ*, *trnC*, and *trnY*) that was different from the above apomorphic tRNA gene block (*trnA*, *trnS* [AGN], *trnN*, *trnE*, and *trnR*). Moreover, this special tRNA string has not been seen in other metazoan mitochondrial genomes in the MitoZoa database^41^. A similar phenomenon also occurred in the mitochondrial genome of *Parhyale hawaiiensis*^42,43^, which had a specific tRNA cluster, *trnI*, *trnA*, *trnN*, *trnR*, and *trnT*, and also without related MitoZoa records. In summary, *Halice* sp. MT-2017 showed particular rearrangements and polarity alternations in its mitochondrion genes, which were seldom seen in most amphipods from other superfamilies.

Further, CREx^44^ was applied to deduce the gene rearrangement scenarios with reference to the ancestral pancrustacean gene pattern. The mitochondrial genome of *Halice* sp. MT-2017 underwent two gene transpositions (*trnN* and *trnV*), one inversion-coupled transposition (*trnR*), three wide-ranging reversals of strands—one involving a gene block unexpectedly composed of 20 genes and the other two including *trnP* and a gene string (*trnS* [UCN], *cytb*, *nad6*, *trnP*, *trnT*, *nad4l*, *nad4*, and *trnH*), respectively—and two complex tandem duplications with subsequent random gene loss (TDRLs) to its present gene order (Fig. S3). The large scale of this inversion event has not been observed in other amphipods; however, it is a characteristic usually seen in the mitochondrial genomes of echinoderms^45^ and metastriate ticks^46,47^. Mechanistically, the long-range reversions in the mitochondrial genome could be well explained by intra-mitochondrial recombination^48,49^, in which both DNA breakage and reconnection are required. A hot spot for the recombination event was thus posited to be relevant to the non-coding AT-rich CR with the origin of replication^50–53^. In the *Halice* sp. MT-2017 mitochondrial genome, moreover, excluding the CR between the two rRNAs, there were intergenic non-coding small spacers spread over 17 locations, ranging from 1 to 100 bp, and comprising 256 bp in total, with a rich AT content of 84.38%. Both the non-coding CR and small spacers were particularly scattered adjacent to genes involved in the above deduced rearrangement courses (Figs 3 and S3) (e.g., the intergenic spacers flanking the gene string from *trnH* to *trnS2* and the gene *trnP*, which were involved in reversal 2 and reversal 3, respectively; the CR between *rrnS* and *rrnL*; and the spacers abutting *trnP*, which were allowed for the TDRL 1). These non-coding regions probably exhibited the traced relics generated from the antecedent gene rearrangement events^38^.

For most metazoan mitochondrial DNA, there was a notable lack of genetic recombinations^20,21^. The significant mitochondrial recombination in this hadal species could be explained as a special approach to escape the consequences of Muller’s ratchet^54,55^. The “ratchet mechanism” states that deleterious mutations would accumulate more easily, especially in a population without recombination. For mitochondrial DNA, this inexorable process would bring about higher and higher mutational levels, to the degree of complete dysfunctionality and even extinction of the genome. Therefore, gene recombination could be a survival strategy for the hadal *Halice* sp. MT-2017 to offset the high mutational rates of mitochondrial DNA^56^.

### Base composition bias of the mitochondrial genome

**Figure 4:**
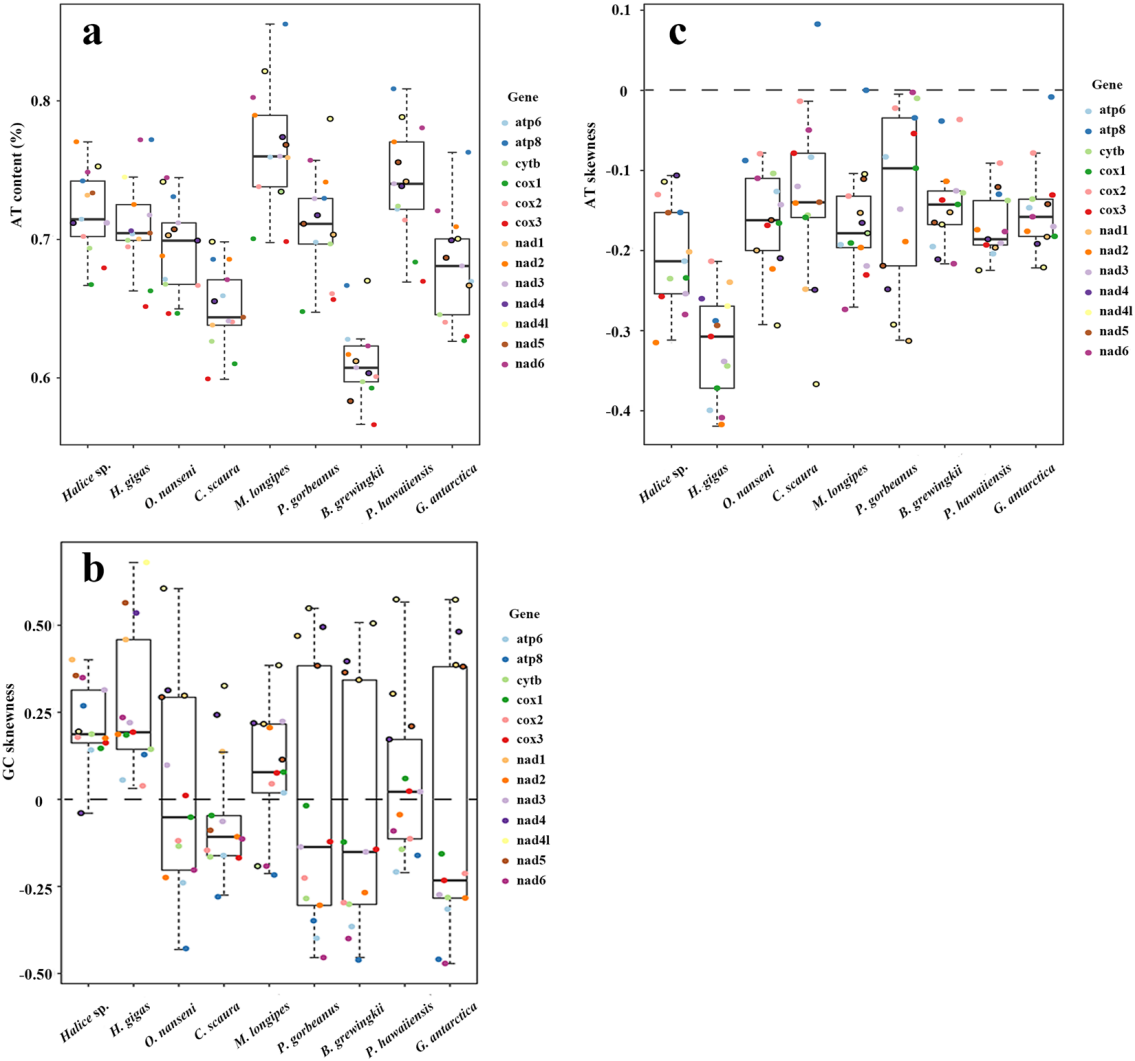
Mitochondrial nucleotide composition of amphipods from different superfamilies. Box plots showed the AT content (**a**), GC skewness **(b)**, and AT skewness **(c)** of 13 PCGs in mitochondrial genomes from nine amphipod species. Each PCG was represented by dots in different colors randomly jittered within the defined box border. The dots for genes encoded on the heavy (or minus) strand were marked by a black circle. Because the *H. gigas* mitochondrial genome had two contigs, the polarities of its PCGs were not discriminated.

The nearly complete mitochondrial genome of *Halice* sp. MT-2017 had an AT content of 74.40%, which was comparable to the typical AT richness in many other amphipod species (Table 2; from 62.24% to 76.03% for complete mitochondrial genomes and from 69.79% to 74.35% for incomplete ones). A comparison of AT contents in 13 PCGs across eight superfamilies showed that there was no distinct pattern observed in both strands (Fig. 4a).

**Table 2:** Nucleotide composition of the mitochondrial genomes in different amphipods.

Species	Length (bp)	A%	T%	G%	C%	A + T%	AT skewness	GC skewness
**Whole mitochondrial genome**								
***Halice*** **sp. MT-2017**	15199	32.84	41.56	15.67	9.93	74.40	−0.117	0.224
***Hirondellea gigas*** **contig1**	8603	25.10	46.80	16.50	11.70	71.90	−0.302	0.170
***Hirondellea gigas*** **contig2**	6984	44.50	28.90	6.10	20.40	73.40	0.213	−0.540
*Onisimus nanseni*	14734	35.03	35.29	11.90	17.78	70.32	−0.004	−0.198
*Caprella scaura*	15079	32.70	33.73	14.54	19.03	66.43	−0.015	−0.134
*Caprella mutica*	15427	33.20	34.75	13.28	18.77	67.95	−0.023	−0.171
*Metacrangonyx longipes*	14113	37.36	38.67	11.56	12.41	76.03	−0.017	−0.035
***Longipodacrangonyx*** **sp**.	12924	33.05	40.29	15.65	11.02	73.34	−0.099	0.174
***Pseudoniphargus gorbeanus***	14178	37.91	33.27	10.25	18.57	71.18	0.065	−0.289
***Pseudoniphargus sorbasiensis***	15460	35.44	34.35	10.03	20.18	69.79	0.016	−0.336
*Brachyuropus grewingkii*	17118	31.22	31.02	13.07	24.68	62.24	0.003	−0.307
*Eulimnogammarus vittatus*	15534	33.22	34.20	12.68	19.90	67.42	−0.015	−0.222
***Parhyale hawaiiensis***	14301	36.23	38.12	11.18	14.47	74.35	−0.025	−0.128
*Gondogeneia antarctica*	18424	34.85	35.29	10.61	19.26	70.13	−0.006	−0.290
**Protein coding genes**								
*Halice* sp. MT-2017	11043	28.45	43.00	17.13	11.42	71.45	−0.204	0.200
*Hirondellea gigas*	11049	23.80	46.29	19.42	10.48	70.10	−0.321	0.299
*Onisimus nanseni*	11049	28.66	39.90	16.34	15.10	68.57	−0.164	0.039
*Caprella scaura*	11004	27.34	36.92	17.10	18.63	64.27	−0.149	−0.043
*Caprella mutica*	10998	27.88	38.12	15.53	18.48	65.99	−0.155	−0.087
*Metacrangonyx longipes*	11082	31.28	44.08	13.33	11.31	75.37	−0.170	0.082
*Longipodacrangonyx* sp	11091	29.92	42.72	14.31	13.06	72.64	−0.176	0.046
*Pseudoniphargus gorbeanus*	11025	29.90	40.16	15.42	14.51	70.07	−0.146	0.030
*Pseudoniphargus sorbasiensis*	11028	28.18	39.35	16.47	16.00	67.53	−0.165	0.014
*Brachyuropus grewingkii*	11064	25.62	34.62	19.79	19.97	60.24	−0.149	−0.004
*Eulimnogammarus vittatus*	11058	28.11	37.51	17.43	16.96	65.62	−0.143	0.014
*Parhyale hawaiiensis*	11040	30.51	42.68	14.13	12.68	73.19	−0.166	0.054
*Gondogeneia antarctica*	10956	28.26	38.77	16.40	16.57	67.03	−0.157	−0.005
tRNA								
*Halice* sp. MT-2017	1401/22	38.83	41.61	11.71	7.85	80.44	−0.035	0.197
***Hirondellea gigas***	1280/21	35.31	38.83	15.08	10.78	74.14	−0.047	0.166
*Onisimus nanseni*	1396/22	37.54	35.53	14.26	12.68	73.07	0.027	0.059
*Caprella scaura*	1315/22	35.21	36.12	16.05	12.62	71.33	−0.013	0.119
*Caprella mutica*	1338/22	36.47	35.58	15.10	12.86	72.05	0.012	0.080
*Metacrangonyx longipes*	1300/22	40.69	37.54	12.85	8.92	78.23	0.040	0.180
***Longipodacrangonyx*** **sp**.	1250/21	39.20	37.20	13.84	9.76	76.40	0.026	0.173
*Pseudoniphargus gorbeanus*	1307/22	36.50	34.97	15.61	12.93	71.46	0.021	0.094
*Pseudoniphargus sorbasiensis*	1320/22	36.67	33.71	16.29	13.33	70.38	0.042	0.100
*Brachyuropus grewingkii*	1304/22	33.28	32.13	18.71	15.87	65.41	0.018	0.082
*Eulimnogammarus vittatus*	1373/23	34.38	32.92	18.06	14.64	67.30	0.022	0.105
*Parhyale hawaiiensis*	1360/22	37.87	37.79	14.04	10.29	75.66	0.001	0.154
*Gondogeneia antarctica*	1364/22	35.63	34.02	16.06	14.30	69.65	0.023	0.058
**rRNA**								
*Halice* sp. MT-2017	1796	39.31	39.76	14.03	6.90	79.06	−0.006	0.340
*Hirondellea gigas*	1614	33.15	40.27	19.64	6.94	73.42	−0.097	0.478
*Onisimus nanseni*	1840	37.77	38.48	15.27	8.48	76.25	−0.009	0.286
*Caprella scaura*	1739	35.02	36.75	16.22	12.02	71.77	−0.024	0.149
*Caprella mutica*	1742	34.33	37.94	16.30	11.42	72.27	−0.050	0.176
*Metacrangonyx longipes*	1751	38.26	40.43	13.54	7.77	78.70	−0.028	0.271
***Longipodacrangonyx*** **sp**.	517	40.62	36.56	12.96	9.86	77.18	0.053	0.136
***Pseudoniphargus gorbeanus***	1663	34.06	42.78	15.70	7.46	76.84	−0.114	0.356
*Pseudoniphargus sorbasiensis*	1706	35.52	40.39	16.82	7.27	75.91	−0.064	0.397
*Brachyuropus grewingkii*	1608	30.72	35.63	23.26	10.39	66.36	−0.074	0.383
*Eulimnogammarus vittatus*	1606	33.06	38.23	19.24	9.46	71.30	−0.072	0.341
***Parhyale hawaiiensis***	1555	36.53	41.67	13.95	7.85	78.20	−0.066	0.280
*Gondogeneia antarctica*	1859	35.45	37.44	17.16	9.95	72.89	−0.027	0.266

Note: All statistical values were calculated with the light strand as reference. Bold species names indicated incomplete sequences in the relevant part of the mitochondrial genome. Only the mitochondrial genome of *H. gigas* was represented by two contigs due to its incompleteness. The number of tRNAs is noted after the slash in the sequence length column.

The mitochondrial genome of *Halice* sp. MT-2017 was skewed away from C in favor of G, resulting in a positive value (0.224) for GC skewness, which was opposite to that of most other amphipods, as shown in Table 2. To further explore this reversed direction of skewness, the skew values of 13 PCGs were compared. As shown in Fig. 4b, six superfamilies represented by *Onisimus nanseni*, *Caprella scaura*, *Pseudoniphargus gorbeanus*, *Brachyuropus grewingkii*, *P. hawaiiensis*, and *Gondogeneia antarctica* followed the common pattern of malacostracans^57^, where the genes encoded on the light strand showed negative GC skew values and, conversely, the genes on the heavy strand exhibited positive GC skew values. However, for *Halice* sp. MT-2017 and *M. longipes* (from the family Metacrangonyctidae)^24^ inhabiting subterranean waters^58^, the PCGs on the light strand showed a reversed pattern in which almost all GC skew values were positive, with the exception of *atp8* and *nad6* in *M. longipes*. This GC skew inversion was also detected in *nad4* of *Halice* sp. MT-2017 and *cytb* of *M. longipes* on the heavy strand (Fig. 4b). Regarding AT skewness (Fig. 4c), it was noted that the overall AT skewness for most superfamilies under study was more intensively inclined on the heavy strand than on the light strand. On the contrary, inclinations of AT skewness in *Halice* sp. MT-2017 and *M. longipes* showed a reversed pattern for the two strands, suggesting the reversion of stand bias for these two species. As to the other hadal species, *H. gigas*, which belonged to the same superfamily (Lysianassoidea) as the amphipod *O. nanseni* in the present study, the GC skew values for all of its 13 PCGs were positive; however, this overall pattern did not accord well with that of *O. nanseni* (Fig. 4b). Therefore, there was the possibility that the mitochondrial genome of *H. gigas* had also suffered strand bias reversion during its evolution, although the two unjointed contigs in its mitochondrial DNA impeded our reassurance of this hypothesis. An explanation for the strand bias reversion could be related to the reversal of mitochondrial CR, the inversion of which would change the mutational constraints for the two mitochondrial strands during DNA replication, transcription, or both^59^. CR inversion has been postulated in Metacrangonyctidae^24^ based on the inverted polarity of *trnS* (UCN) and *cytb* near the CR. *Halice* sp. MT-2017 in the present study showed a similar case with inverted *rrnS* and *rrnL* flanking the CR. Based on the analysis in the present study, the taxa showing the reversed pattern did not appear to be phylogenetically clustered or have related ecological habitats; therefore, the reversal of the ordinary strand bias probably occurred independently multiple times during the evolution of amphipods, especially in the relatively ancient superfamilies represented by *Halice* sp. MT-2017 and *M. longipes* (Fig. 2).

### Amino acid and codon usage frequency

**Figure 5:**
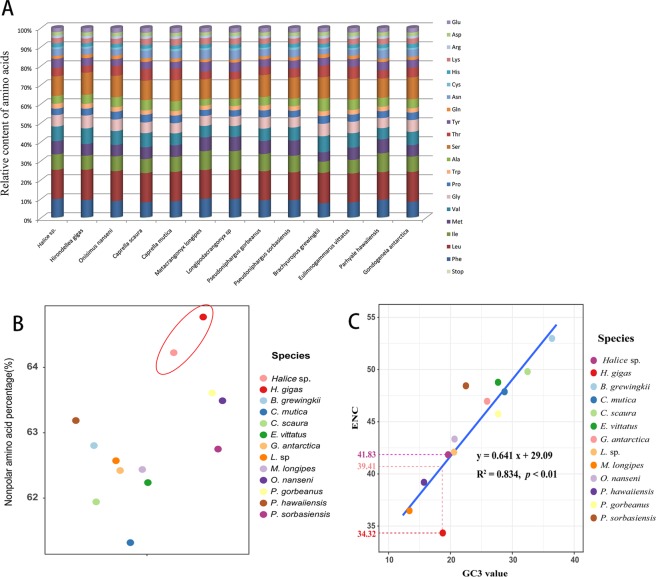
Statistical data for amino acid contents and ENCs within the mitochondrial PCGs of amphipods from different superfamilies. (**A**) Relative amino acid contents within the mitochondrial genome of amphipods from different superfamilies. (**B**) Percentage of nonpolar amino acids in mitochondrial PCGs of different amphipods. The dots representing the two hadal species showed significantly higher values than other dots. (**C**) The relationship between the effective number of codons (ENC) and the GC content at the third codon position (GC3).

There was a total of 3,670 amino acids in the 13 mitochondrial PCGs of *Halice* sp. MT-2017. The amino acid composition of *Halice* sp. MT-2017 was consistent with those of the amphipods from the other seven superfamilies (Fig. 5A), with Leu and Ser the most frequently used amino acids, accounting for approximately a quarter of the amino acids in total, and Cys, Arg, and Glu being rarely used. Nevertheless, there were still small variations in the frequency of each amino acid between different species. Considering that the amino acid composition and properties could have an influence on the function of proteins^60,61,62^, these two parameters were compared between the two hadal species (*Halice* sp. MT-2017 and *H. gigas*) and the other 11 amphipods. Statistical *t*-tests showed that the percentages of non-polar amino acids from the two hadal mitochondrial genomes (64.50 ± 0.39%) were significantly higher than those of the amphipods in shallow water (62.61 ± 0.67%) (Fig. 5B). Accordingly, the composition of the polar uncharged amino acids and charged amino acids were remarkably higher in the non-hadal amphipods than in the hadal amphipods (Table S4). Therefore, polarity seemed to play an important role in protein stability under the conditions of the hadal environment. It has been reported that only tens of atmospheres of pressure would be necessary to cause dysfunction in the protein activity of shallow water species^63^. The fauna in the hadal trench would likely have evolved special mechanisms to cope with the thousands of atmospheres of pressure in the deep sea. At denaturing pressure, membranes or related processes are among the most sensitive to hydrostatic pressure^64^. As the 13 PCGs of mitochondria were all transmembrane proteins embedded in the hydrophobic lipid chains of the membrane^65,66^, the increase in non-polar amino acid content might be conducive to the compaction interaction between the membrane proteins and the lipid chains in the mitochondrial membrane, thus ameliorating the potential of damage caused by the pressure on the membrane. The maintenance of the mitochondrial structure provided a premise for these hadal amphipods to sustain metabolism, growth, and even reproduction, and would be an adaptation agreeable with the extreme environment in the hadal trench.

The codon usage of *Halice* sp. MT-2017 was shown in Table S5; it was consistent with the canonical types of invertebrate mitochondrial codes^67^. All of the 13 PCGs were initiated with the ATN, which was the typical start codon for the metazoans^26^. Regarding the stop codons, 12 of the 13 PCGs used TAN as their termination codon, whereas only *cox2* terminated with a single T. This truncated stop codon was believed to be completed by post-transcriptional polyadenylation^68^. The most frequently used codons in *Halice* sp. MT-2017 included TTA (Leu, 9.94%), TTT (Phe, 9.26%), ATT (Ile, 7.14%), and ATA (Met, 5.57%). Moreover, a preference for these four codons was also observed in other non-hadal species (Table S4). In *H. gigas*, apart from the TTT (Phe, 9.04%), TTA (Leu, 7.98%), and ATT (Ile, 7.20%) mentioned above, TTG (4.75%) encoding Leu was the codon also used more frequently, which was not found in other amphipods. Overall, there was a bias in favor of AT-rich codons in all of the currently studied amphipods, which was still notable for other arthropods^69^.

Nonrandom usage of synonymous codons is a common phenomenon in nature^70^. The effective number of codons (ENC) is a simple metric of the synonymous codon usage bias, ranging from 20, if all the amino acids are encoded by only one codon, to 62, when all the synonymous codons are equally used^71^. For all amphipods in the present study, the ENC values ranged from 34.32 (*H. gigas* in Lysianassoidea) to 52.97 (*B. grewingkii* in Gammaroidea). The ENC of *Halice* sp. MT-2017 was 41.83, indicating that approximately two-thirds of the possible codons were employed effectively in its mitochondrial genome. Although the ENCs were species-specific among all amphipods under study, there were two codon usage patterns when referring to their relationship with the GC contents at the third synonymous codon position (GC3) (Fig. 5C). The first pattern showed a linear correlation between the ENC values and the GC3s (*R*^2^ = 0.834, *p* < 0.01). In this pattern, the synonymous codon usage was associated with the G + C content of the mitochondrial DNA and the ENCs reflected the species-specific mutational bias around different mitochondrial genomes^72^. Most amphipods under study displayed this pattern. However, some species showed a significant deviation from the linear association, such as *P. gorbeanus* and *H. gigas* in Fig. 5C. The number of the effectively used codons (34.32) in *H. gigas* was lower than that estimated by the regression formula (39.41), indicating a relatively strong bias of codon usage in *H. gigas*. The codon usage bias of *H. gigas* reflected the natural selection for certain codons, through which highly expressed genes exhibited a greater degree of preference for a particular subset of codons than the genes that were less expressed^71^. In summary, the diverged codon usage patterns shown in the two hadal species indicated that mutation pressure and natural selection imposed relatively different forces during their evolutions^73^.

### Transfer RNAs and Ribosomal RNAs

As shown in other metazoans, 22 tRNAs were identified in *Halice* sp. MT-2017 mitochondrial DNA, ranging from 55 bp (*trnE* [UUC]) to 69 bp (*trnK* [UUU]) to make 1,401 bp in total (Table 1). Of the 22 tRNAs, 14 genes were encoded on the light strand, whereas the remainders were on the heavy strand. Compared to the tRNAs of other amphipods, the AT content for *Halice* sp. MT-2017 was the highest (80.44%). The negative AT skews (−0.035 and −0.047) of tRNAs for *Halice* sp. MT-2017 and *H. gigas* were opposite to most amphipods with positive AT skew values (Table 2). The reversed strand bias of the AT skew probably resulted from the inversion of the polarity of some tRNA genes or the related CR, a case similar to the reversion of PCGs skewness. The secondary structures of tRNAs in *Halice* sp. MT-2017 were illustrated in Fig. S4. A total of 20 out of 22 tRNAs in *Halice* sp. MT-2017 could be folded into a complete cloverleaf structure, although there were losses of dihydrouridine (DHU) arms in *trnS* (UCU) and *trnV*. Disappearance of the DHU domain in *trnS* (UCN) was common in almost all of the metazoan mitochondrial genomes^24^, and the loss of the DHU arm in *trnV* could be observed in many other amphipods as well, such as *B. grewingkii*, *E. cyaneus*, *Acanthogammarus victorii*, and *Garjajewia cabanisii*^23^. The aberrant structures of tRNA in the mitochondrial genome is not unusual in crustaceans^47,74,75–77^, which can be explained by the selection bias towards a minimal mitochondrial genome^78^ or the process of replication slippage^79^.

The AT content of rRNA genes for *Halice* sp. MT-2017 was 79.06% (Table 2), which was the highest value compared to other complete rRNAs of the amphipods under study. The AT and GC skew values were negative (−0.006) and positive (0.340), respectively, which was analogous to that observed for other amphipods (Table 2). The *rrnS* and *rrnL* were 735 bp and 1,061 bp in length, respectively, with 1,796 bp in total (Table 1), and the two rRNA genes were located on the light strand, which was different from the positions of rRNAs observed for other amphipods on the heavy strand.

### Evolutionary rate estimation

The mitochondrial PCGs of amphipods from different taxonomic classifications and ecological environments were used to estimate the non-synonymous/synonymous substitution (dN/dS) ratios under the branch model, assuming the rate consistency along each codon site in the branch^80^. The results showed that all of the evolutionary rates (dN/dS ratios) referring to the whole mitochondrial genome under study were less than one, indicating that the function of the mitochondrial DNA was well-maintained during evolution. Moreover, it was noteworthy that the hadal species (*Halice* sp. MT-2017 and *H. gigas*) demonstrated smallest evolutionary rate values than did the amphipods in other habitats (Table 3). The slower evolutionary rate of mitochondrial genomes in deep-sea species has also been discovered in isopods^81^, which may be related to the relatively constant environment in the deep sea. To explore the genes making contributions to the overall slower evolutionary rates of these two hadal mitochondrial genomes, the dN/dS ratios were calculated in 13 PCGs separately. The results showed that *nad4* and *cox2* had lower evolutionary rates for both hadal amphipods; *nad6* exhibited a lower evolutionary rate for *Halice* sp. MT-2017, and *cox3*, *nad4l*, and *nad5* showed lower evolutionary rates for *H. gigas* (Table S6). The slower evolutionary rates for these genes indicated that they were under a stronger purification selection^80^, which was critical in removing the disadvantageous mutations and maintaining mitochondrial gene functions^22^.

**Table 3:** Values for mutations and dN/dS ratios of 13 mitochondrial tandem genes for amphipods from different taxonomic classification and ecological habitats.

Superfamily	Species	Ecological features	dN	dS	dN/dS
Pardaliscidae	*Halice* sp. MT-2017	Mariana Trench at 10908 m in this study	0.2155	6.5386	0.0330
Lysianassoidea	*Hirondellea gigas*	Hadal trench, distributed deeper than 7000 m up to over 10,000 m	0.1225	4.5454	0.0269
Lysianassoidea	*Onisimus nanseni*	Arctic pack ice	0.1579	2.9702	0.0532
Talitroidea	*Parhyale hawaiiensis*	Shallow sea	0.1192	3.4176	0.0349
Gammaroidea	*Brachyuropus grewingkii*	Estuary of Buguldeyka river, 100–1380 m	0.1690	2.7751	0.0609
Caprelloidea	*Caprella scaura*	Shallow sea	0.2748	2.6342	0.1043
Hadzioidea	*Metacrangonyx longipes*	Subterranean waters	0.1897	4.6340	0.0409
Allocrangonyctoidea	*Pseudoniphargus gorbeanus*	Subterranean waters	0.1648	3.1928	0.0516
Calliopioidea	*Gondogeneia antarctica*	Intertidal rocky shore in Antarctica	0.1709	3.0341	0.0563

## Materials and Methods

### Sampling and DNA extraction

Individuals of *Halice* sp. MT-2017 (Fig. 1) for DNA extraction were collected from the Mariana Trench (E142°11.4152′, N11°19.4990′) at a depth of 10,908 m in March 2017. Other stations where *Halice* sp. MT-2017 could be discovered are listed in Table S1. Sampling of *Halice* sp. MT-2017 was performed by trapping with bait in a modified sampler installed on the lander (the description of this modified device will be reported in a separate paper). Specimens for mitochondrial genome analysis were frozen in liquid nitrogen and stored at −80 °C until subsequent use. Total genomic DNA was prepared from the head of *Halice* sp. MT-2017 using an E.Z.N.A.^®^ Tissue DNA Kit (OMEGA, Wuhan, China) according to the manufacturer’s instructions. The concentration of total isolated DNA was determined with a Qubit Fluorometer (Thermo Scientific, USA) and the quality of extracted DNA was visualized by electrophoresis on 1% agarose gel stained with SYBR ^®^ Safe DNA gel stain (Thermo Scientific, USA).

### Genome sequencing

A paired-end library with an insert size of 300 bp was prepared with total genomic DNA using the TruSeq DNA Sample Prep Kit (Illumina, USA). The above library was sequenced by an Illumina HiSeq2000 (2 × 150 bp paired-end reads) (Illumina, USA).

### Assembly of the mitochondrial genome and PCR verification

Adapters and parts with a quality score below 15 were removed from raw reads with a Trimmomatic 0.36 tool^82^. The clean reads were assembled using SPAdes 3.11.0 assembler^83^ with default parameters. The assembled contigs were blasted against the mitochondrial DNA sequence of *M. longipes* (GenBank Accession No.: AM944817) to extract mitochondrial sequences using the BLAST tool^84^ with the sequence of amphipod *M. longipes* (accession no.: AM944817) as a reference. The average coverage depth for the obtained mitochondrial genome sequence was calculated using Bowtie2 2.2.4^85^ by mapping the clean reads to the extracted contigs. Visualization of the alignment file was realized using Tablet 1.17.08.17^86^. The accuracy of the assembly of mitochondrial genome was verified by PCR. Primers to amplify the mitochondrial genome are listed in Table S7. The experimental conditions for the PCR were taken from Shen *et al*.^81^.

### Genome sequence annotation and analysis

The mitochondrial genome was preliminarily annotated by the MITOS webserver (http://mitos.bioinf.uni-leipzig.de/index.py)^87^. Boundaries of the 13 PCGs and 2 rRNAs were determined by alignment with the homologous genes of other amphipods. Transfer RNA genes and their secondaries were predicted by the MiTFi^88^ model in the MITOS pipeline^87^, and further confirmed by the ARWEN 1.2.3.c^89^ and tRNAscan-SE 1.21^90^ software programs. The MitoZoa 2.0 database was used to compare the gene order of *Halice* sp. to those of other species^42^. Gene rearrangement scenarios were deduced by detecting strong interval trees on the CREx webserver (http://pacosy.informatik.uni-leipzig.de/crex/)^44^. The gene order of *Halice* sp. MT-2017 was compared to those of other amphipods or to the putative ancestral pancrustacean ground pattern^91^. The nucleotide composition was computed by the DNAMAN sequence analysis software program (Lynnon BioSoft, Vaudreuil, Canada). The skew values of AT and GC were calculated according to following formulae: AT skew = (A − T)/(A + T) and GC skew = (G − C)/(G + C), in which A, T, G, and C were the contents of four bases^92^. The codon usage was analyzed using the Sequence Manipulation Suite^93^. The relative synonymous codon usage was calculated with MEGA 6.0^94^. The ENC^71^ was determined using the INCA 2.1 software program^95^. The percentage of each amino acid was calculated by summarizing all of its corresponding codons. A two-tailed *t-test* was performed using the ‘*t.test*’ function in R software (3.5.1) to calculate differences and the significant levels (*p*-value) for amino acid contents, as well as their corresponding property groups, between the deep-sea species (*Halice* sp. MT-2017 and *H. gigas*) and the amphipods from shallower waters (listed in Table 3).

### Substitution rate estimation

To estimate the dN/dS ratios, standard branch models were performed on the 13 concatenated mitochondrial PCGs and 13 separate PCGs, respectively, with the ‘*codeml*’ program in the PAML 4.7 software package. A “free-ratio” model was set and the ambiguous characters and the alignment gaps were removed^80^.

### Phylogenetic inference

In the aspect of DNA barcoding, eight available partial *cox1* (cytochrome c oxidase subunit I) sequences of Pardaliscidae in Genbank were used to illustrate the taxonomic placement of the hadal *Halice* sp. MT-2017 specimens collected below 10,000 m. Eight *cox1* sequences from other closely related families were also included in the tree construction. Related accession numbers are listed in Table S8. All of the sequences were aligned using MUSCLE 3.8.31^96^. Based on the well-aligned 603 nt sequence, Bayesian and maximum likelihood methods were used to construct phylogenetic trees with MrBayes 3.2.6^97^ and RaxmlGUI 1.3^98^ respectively, using the GTR + G + I model as recommended by jModelTest 2^99^. Four independent runs of four MCMC chains were performed for Bayesian analysis. Chains were run for five million generations, and the first 25% of generations were discarded as burn-in. The node stability of the maximum likelihood tree was assessed with 1,000 bootstrap replicates.

Phylogenetic analyses were also performed based on the mitochondrial genomes of *Halice* sp. MT-2017 and those of other 12 amphipods belonging to the seven distinct superfamilies available in GenBank, with four species of isopods used as outgroups. Detailed information of the sequences used was shown in Table S8. Both nucleotide and amino acid sequences of the 13 PCGs were aligned using MUSCLE 3.8.31^96^ separately. Removing the poorly aligned regions and concatenated conserved sequences were performed using Gblocks 0.91b^100^ with default stringent parameters. After being trimmed by Gblocks, the remaining nucleotide and amino acid datasets consisted of 9,732 nt and 2,816 aa, respectively. Phylogenetic analysis for each dataset was carried out using the ML method. Regarding the nucleotide sequences, the GTR + G + I model was selected by jModelTest 2^99^. For the amino acid dataset, the MtArt + G + I + F model was selected by ProtTest 3.4^101^. ML analysis was carried out using RaxmlGUI 1.3^98^ for the nucleotide dataset and PhyML 3.0^102^ for the amino acid dataset, both of which were conducted with 1,000 bootstrap replicates. Genetic distances between clades were computed using Mega 6.0^94^ with the *p*-distance mode for both *cox1* and 13 concatenated PCGs sequences.

There was a lack of an appropriate fossil record for the calibration of a molecular clock with regard to amphipods^103^; instead, geological events for molecular calibration were used. The formation of the shallow lake, Lake Baikal, occurred approximately 27–35 Mya, and the molecular study revealed that the main Baikal amphipods (*Eulimnogammarus vittatus* and *B. grewingkii* in the present study) occurred at a comparable time to the formation of Lake Baikal^104,105^. BEAST v1.8.4^106^ was implemented to estimate divergence times. An uncorrelated relaxed lognormal clock with the GTR + I + G substitution model was used, and a Yule process was set to the tree prior. A normal distribution was applied to the tree calibration node and the most recent common ancestor of Baikalian amphipods was set at 30 ± 2 Mya. Following a burn-in of the initial 50% of cycles, divergence times were sampled once every 1,000 generations from 600 million MCMC iterations. The sampled trees and the associated 95% highest posterior density distributions around the estimated node ages were annotated in TreeAnnotator v1.8.4 (BEAST software). Visualization of the tree was realized in FigTree v1.4.3. The effective sample sizes (ESSs) were used for determining the Bayesian statistical significance of each parameter in TRACER v1.5 (ESS > 200)^107^.

## Supplementary information

Supplementary information
