# Supplementary material for: Characterization of the mitochondrial genome of an ancient amphipod Halice sp. MT-2017 (Pardaliscidae) from 10,908 m in the Mariana Trench
Source: Sci Rep. 2019 Feb 22;9:2610. doi: 10.1038/s41598-019-38735-z (PMC6385184; doi:10.1038/s41598-019-38735-z)
Supplement: Supplementary file 1 — Supplementary information [file 41598_2019_38735_MOESM1_ESM.docx]

**Characterization of the mitochondrial genome of an ancient amphipod *Halice* sp. MT-2017 (Pardaliscidae) from 10,908 m in the Mariana Trench**

Jun-yuan Li ^1,2^, Cong Zeng ^1,3^, Guo-yong Yan ^1,2^ & Li-sheng He ^1 *^

^1^ Institute of Deep-sea Science and Engineering, Chinese Academy of Sciences, Sanya, Hainan, China

^2^ University of Chinese Academy of Sciences, Beijing, China

^3^ Hunan Agricultural University, Changsha, Hunan, China

^*^Correspondence and requests for materials should be addressed to L.-S.H. (email: he-lisheng@idsse.ac.cn)

**
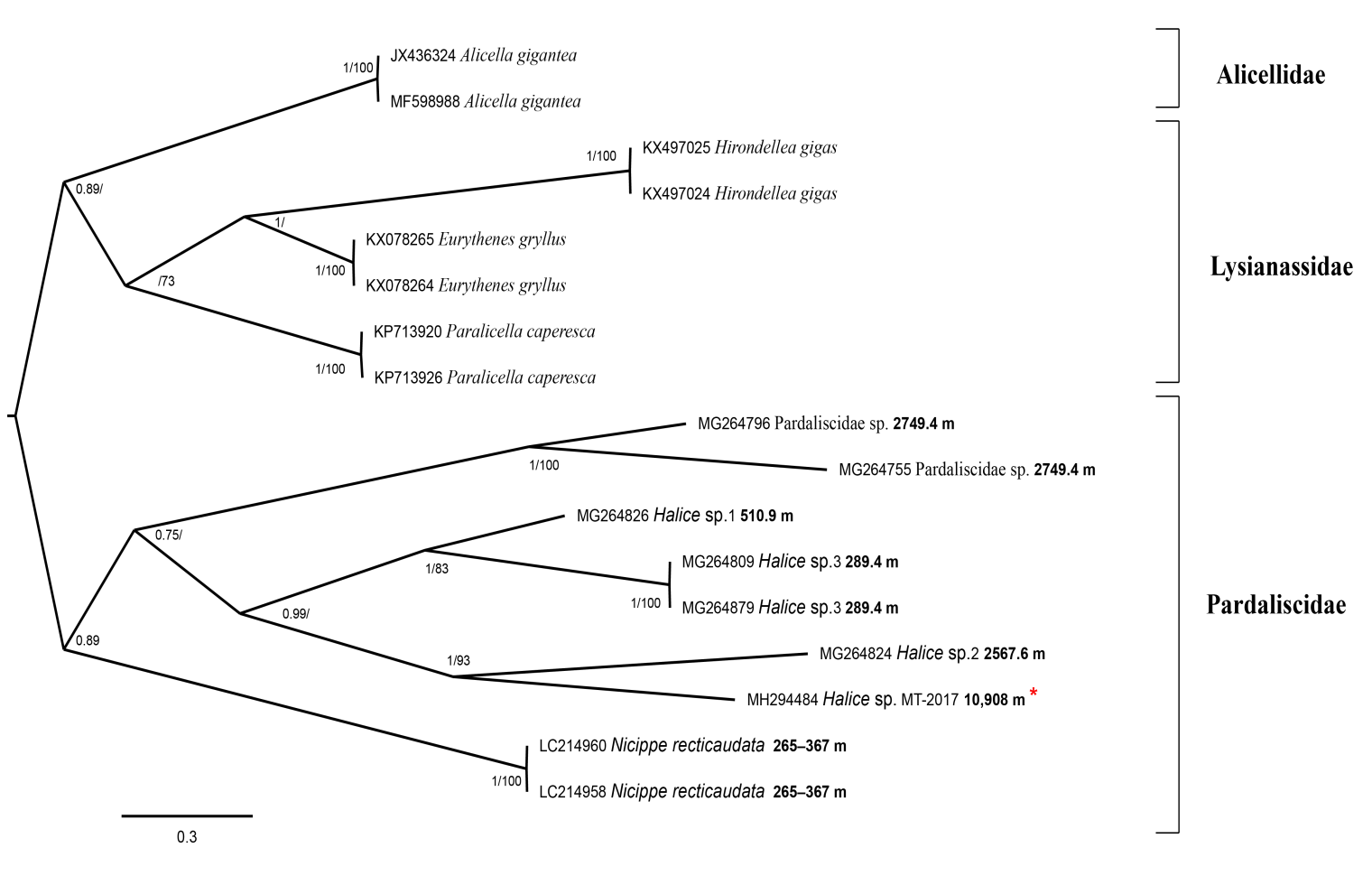
Figure S1. Phylogenetic analysis based on *cox1* partial sequences with maximum likelihood and Bayesian methods.** * denote the sequences produced in this study. Only bootstrap percentages greater than 70% and posterior possibility greater than 0.7 are shown. The scale bar represents 30% sequence divergence. The accession numbers of the sequences used in the phylogenetic analysis are listed in Supplementary Table S8.


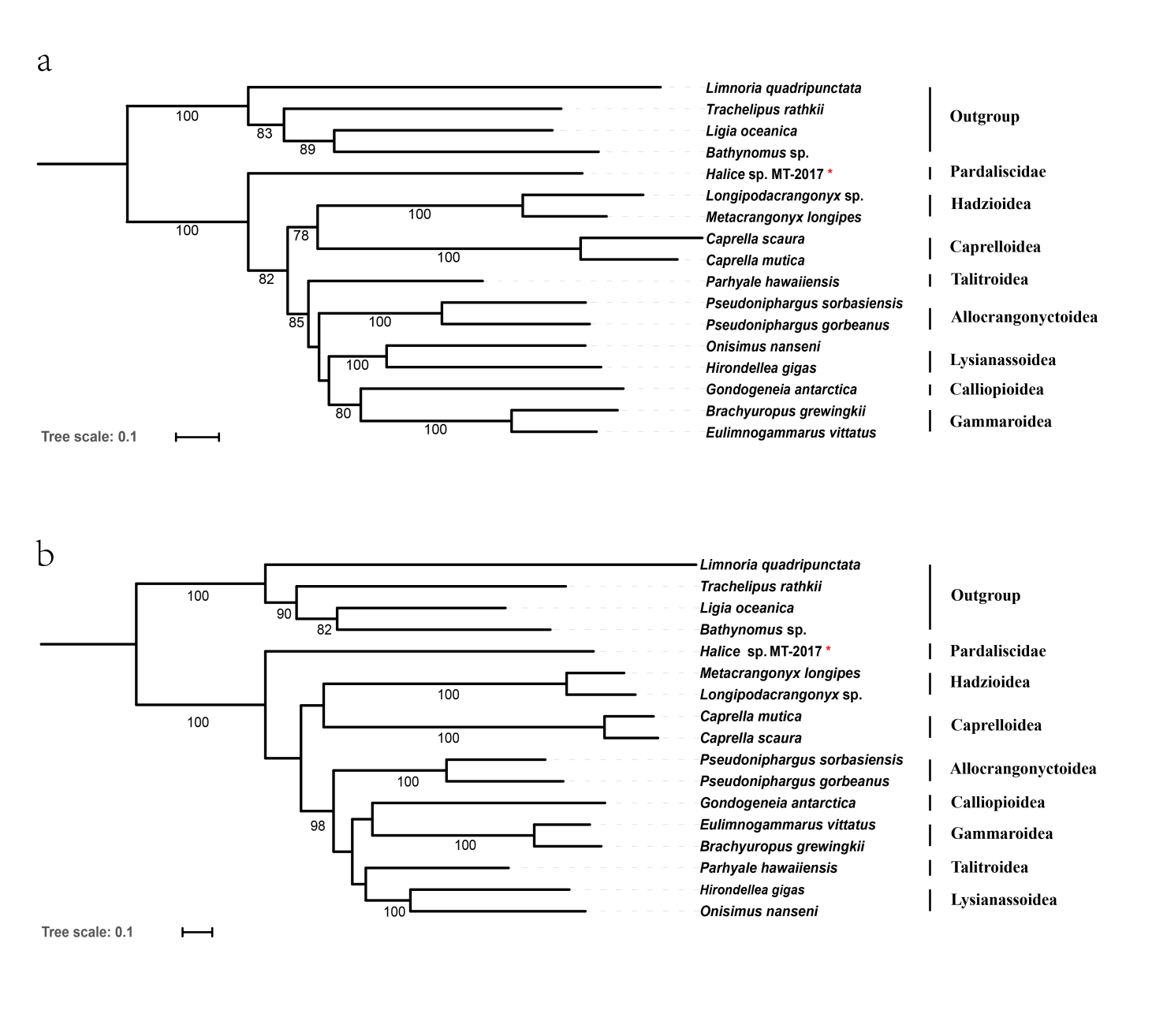


**Figure S2 Phylogenetic trees of the amphipod relationships based on the nucleotide (a) and amino acid (b) datasets for the 13 concatenated** **PCGs in mitochondrial genomes.** * denote the taxa collected from the hadal trench in the present study. Only bootstrap percentages greater than 70% are shown. Four sequences from Isopoda were used as outgroup. The scale bar represents 10% sequence divergence. The accession numbers of the sequences used in the phylogenetic analysis are listed in Supplementary Table S8.

**
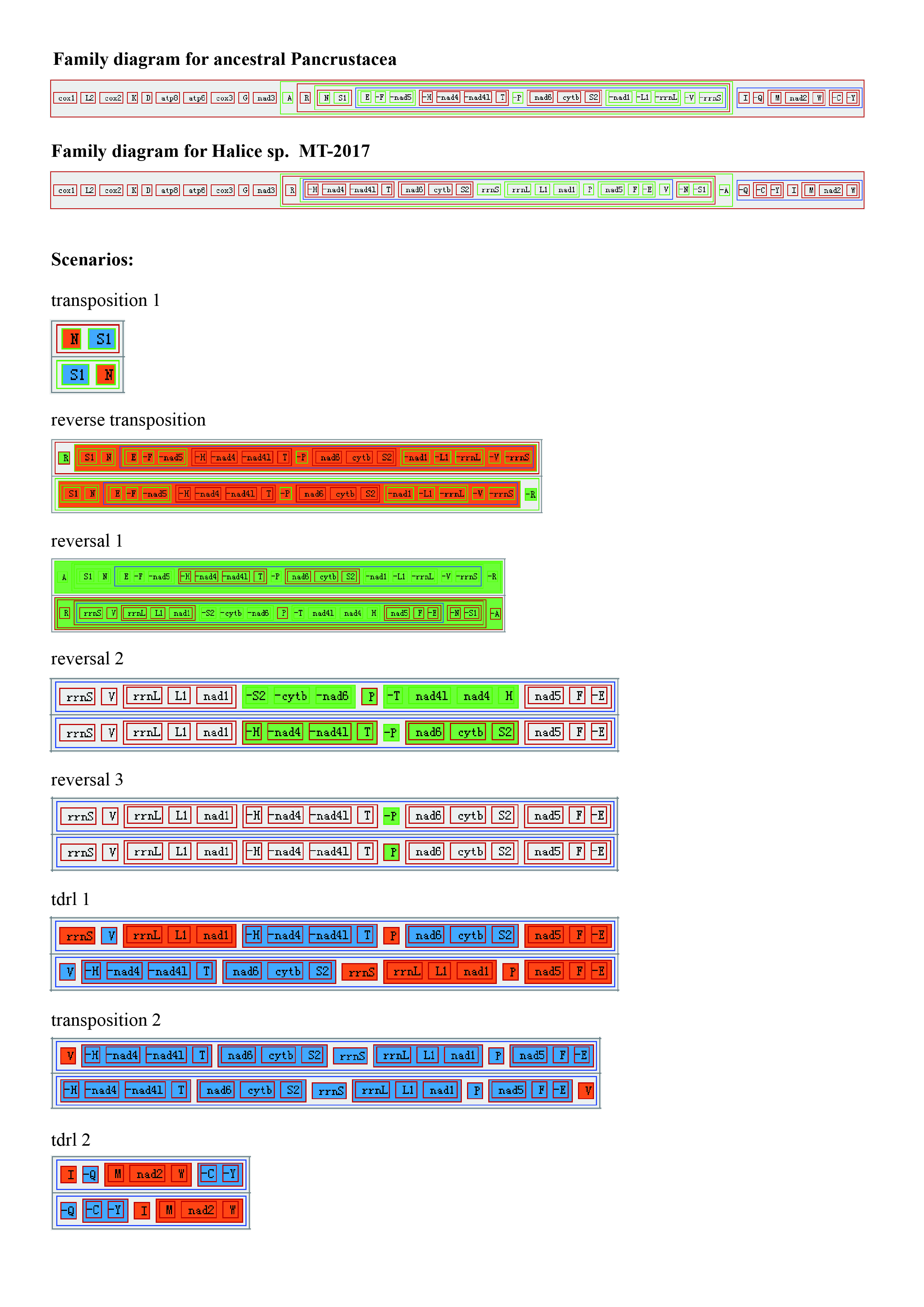
Figure S3. Scenarios deduced for the process of transformation from the ancestral pancrustacean gene order to the extant gene order for *Halice* sp. MT-2017 mitochondrial genome by detection of strong interval trees.**

**
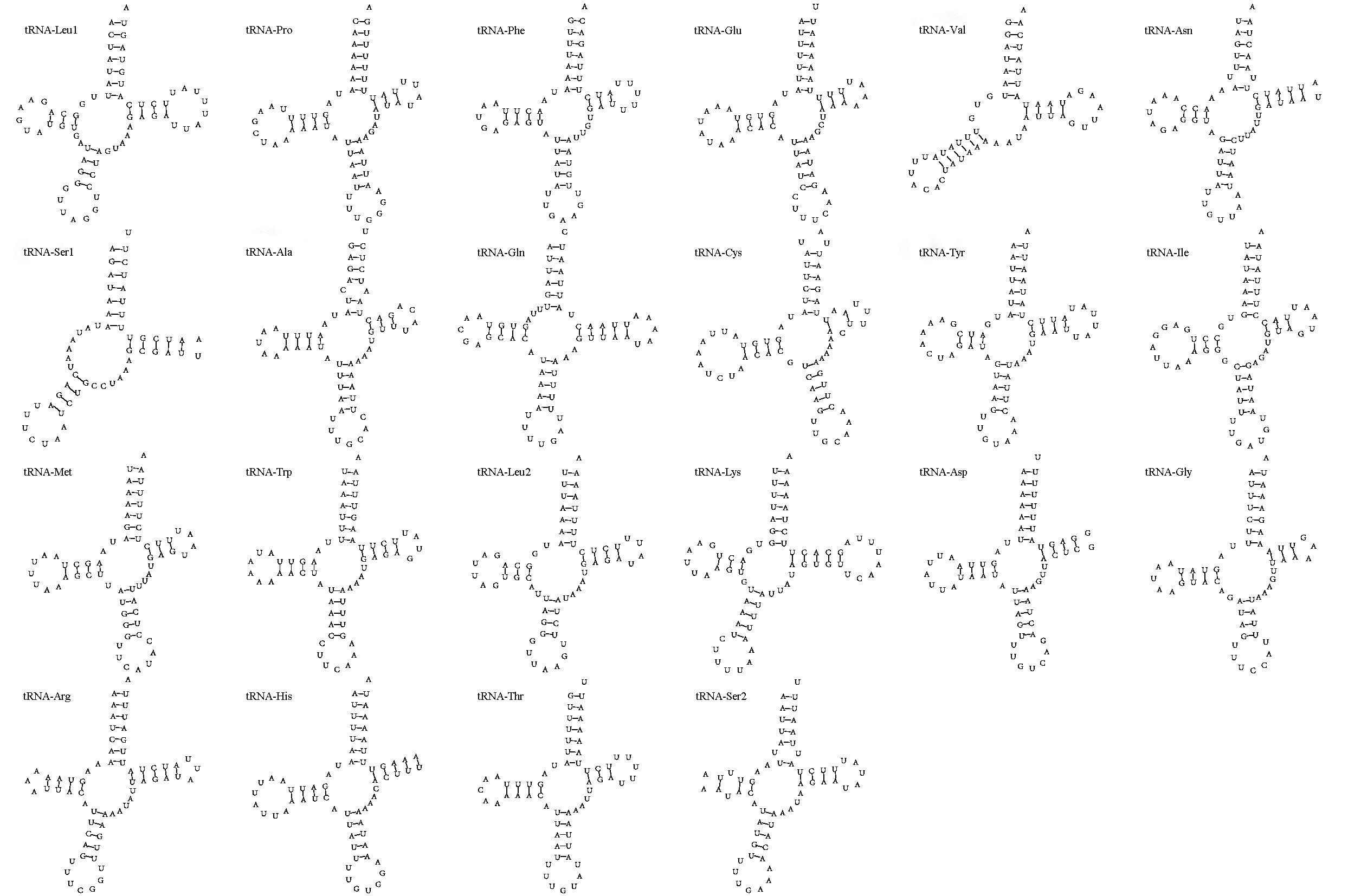
**

**Figure S4. Putative secondary structures for the 22 transfer RNAs in mitochondrial genome of *Halice* sp. MT-2017.**

| **Table S1. Information of the sampling stations and sampling quantities of *Halice* sp. MT-2017and *H. gigas.*** | | | | |
| --- | --- | --- | --- | --- |
| **Station locality** | **Depth (m)** | **no. of *Halice* sp. MT-2017**  **(**body length < 1cm**)** | **no. of *H.gigas***  **(** 2-5cm for samples  below 10,000m**)** | ***p* value of t-test*(Halice* sp*.* vs *H. gigas)*** |
| E 142° 11.3161', N 11° 19.5000' | 10,911 | 15 | 7 | 0.31 |
| E 142° 11.6124', N 11° 19.6171' | 10,909 | 35 | 16 |  |
| E 142° 11.4152', N 11° 19.4990' | 10,908 | 10 | 21 |  |
| E 142° 12.1146', N 11° 19.9236' | 10,904 | 30 | 5 |  |
| E 142° 12.1356', N 11° 19.7940' | 10,903 | 7 | 8 |  |
| E 142° 11.9583', N 11° 19.7942' | 10,901 | 3 | 5 |  |
| E 142° 11.3760', N 11° 19.5341' | 10,899 | 6 | 10 |  |
| E 142° 11.8705', N 11° 19.7984' | 10,893 | 8 | 2 |  |
| E 142° 10.2738', N 11° 25.0148' | 8,920 | 0 | 23 | <0.05 * |
| E 141° 35.0185', N 10° 58.8383' | 8,226 | 0 | ～400 |  |
| E 142° 09.4685', N 11° 08.1118' | 8,196 | 0 | 342 |  |
| E 142° 09.4188', N 11° 07.9529' | 8,152 | 0 | 220 |  |
| E 141° 56.1962', N 10° 59.6724' | 7,125 | 0 | ～1000 |  |

Note: Analyses of statistic significance were performed between the quantities of *Halice* sp. MT-2017 and *H. gigas* with respect to sampling stations deeper than 10,000 m and from 7,125 m to 9,000 m respectively. * indicates the difference of the compared groups reaches significant level.

| **Table S2. Interspecific variations in *Halice* MT-2017 measured by p-distances (%) based on partial *cox1* gene.** | | | |
| --- | --- | --- | --- |
|  | **[1] (%)** | **[2] (%)** | **[3] (%)** |
| **[1] *Halice* sp. MT-2017** |  |  |  |
| **[2] *Halice* sp. 1** | 27.03 |  |  |
| **[3] *Halice* sp. 2** | 26.87 | 28.69 |  |
| **[4] *Halice* sp. 3** | 27.36 | 22.55 | 27.53 |

| **Table S3. Intergroup variations in Amphipoda measured by p-distances (%) based on 13 concatenated protein coding genes of mitochondrial genome.** | | | | | | | |
| --- | --- | --- | --- | --- | --- | --- | --- |
|  | **[1] (%)** | **[2] (%)** | **[3] (%)** | **[4] (%)** | **[5] (%)** | **[6] (%)** | **[7] (%)** |
| **[1] Pardaliscidae** |  |  |  |  |  |  |  |
| **[2] Lysianassoidea** | 37.24 |  |  |  |  |  |  |
| **[3] Talitroidea** | 35.32 | 32.43* |  |  |  |  |  |
| **[4] Allocrangonyctoidea** | 38.65^#^ | 36.02 | 33.11 |  |  |  |  |
| **[5] Hadzioidea** | 37.93 | 36.76 | 34.08 | 37.73 |  |  |  |
| **[6] Calliopioidea** | 40.32 | 36.25 | 34.18 | 36.78 | 38.20 |  |  |
| **[7] Caprelloidea** | 41.52^#^ | 39.83 | 37.82 | 39.65 | 40.28* | 39.76 |  |
| **[8] Gammaroidea** | 40.25 | 36.95 | 34.63 | 36.98 | 39.54 | 37.31 | 40.46 |
| Note: * the genetic boundaries of the superfamily defined by inter-group distances of seven amphipod superfamilies. # the maximal and minimal genetic distances of Pardaliscidae to the taxa from other superfamilies. | | | | | | | |

| **Table S4.** **The proportions (%) of amino acid of mitochondrial PCGs from amphipods of different superfamilies.** | | | | | | | | | | | | | | | |
| --- | --- | --- | --- | --- | --- | --- | --- | --- | --- | --- | --- | --- | --- | --- | --- |
| **Category of amino acids** | **Amino acids** | ***H.* sp.** | ***H. gigas*** | ***O. nanseni*** | ***C. scaura*** | ***C. mutica*** | ***M. longipes*** | ***L.* sp** | ***P. gorbeanus*** | ***P. sorbasiensis*** | ***B. grewingkii*** | ***E. vittatus*** | ***P. hawaiiensis*** | ***G. antarctica*** | ***P value*** |
| **Non-polar amino acids** | **Phe** | 9.75 | 9.16 | 8.56 | 7.93 | 8.46 | 9.83 | 9.69 | 8.98 | 9.09 | 7.43 | 7.92 | 9.28 | 8.22 | 0.2 |
|  | **Leu** | 15.4 | 16.02 | 15.89 | 15.49 | 15.58 | 15.19 | 15.31 | 15.4 | 14.69 | 16.14 | 15.33 | 14.61 | 15.8 | 0.41 |
|  | **Ile** | 8.15 | 7.38 | 7.79 | 7.36 | 7.88 | 9.94 | 9.99 | 9.09 | 8.77 | 5.88 | 7.11 | 9.99 | 8.08 | 0.56 |
|  | **Met** | 7 | 6.19 | 6.08 | 6.24 | 6.19 | 7.33 | 7.49 | 6.93 | 8.03 | 4.98 | 6.51 | 7.37 | 6.07 | 0.92 |
|  | **Val** | 7.71 | 8.28 | 7.33 | 7.5 | 6.3 | 6.03 | 5.81 | 6.5 | 6.94 | 8.49 | 7.65 | 5.93 | 7.06 | 0.1 |
|  | **Gly** | 6.05 | 6.81 | 6.1 | 5.64 | 5.37 | 5.27 | 4.97 | 5.95 | 5.3 | 6.56 | 6.02 | 5.27 | 6.21 | 0.08 |
|  | **Pro** | 3.41 | 3.6 | 4.11 | 4.08 | 4.02 | 3.15 | 3.31 | 3.66 | 3.5 | 4.05 | 3.78 | 3.63 | 3.93 | 0.33 |
|  | **Trp^*^** | 2.62 | 2.56 | 2.4 | 2.3 | 2.38 | 2.28 | 2.25 | 2.57 | 2.4 | 2.53 | 2.45 | 2.4 | 2.25 | **0.02** |
|  | **Ala** | 4.14 | 4.77 | 5.23 | 5.42 | 5.15 | 3.4 | 3.75 | 4.53 | 4.04 | 6.75 | 5.47 | 4.7 | 4.78 | 0.59 |
|  | **Summation^**^** | 64.22 | 64.77 | 63.49 | 61.94 | 61.32 | 62.43 | 62.57 | 63.61 | 62.75 | 62.8 | 62.24 | 63.19 | 62.41 | **0** |
| **Polar-uncharged amino acids** | **Ser** | 10.3 | 11.66 | 11.31 | 10.26 | 11.11 | 10.49 | 10.31 | 11.55 | 11.11 | 11.24 | 11 | 10.19 | 11.57 | 0.9 |
|  | **Thr** | 4.5 | 3.73 | 5.31 | 6.21 | 6.13 | 4.13 | 3.99 | 4.29 | 4.72 | 6.18 | 6.23 | 4.34 | 5.19 | 0.15 |
|  | **Tyr** | 4.77 | 4.01 | 3.51 | 3.72 | 3.56 | 5 | 4.94 | 4.04 | 4.26 | 4 | 4.14 | 4.4 | 4.01 | 0.54 |
|  | **Gln** | 1.5 | 1.77 | 1.85 | 1.83 | 1.72 | 1.44 | 1.55 | 1.64 | 1.72 | 1.9 | 1.85 | 1.58 | 1.62 | 0.58 |
|  | **Asn** | 3.62 | 3.19 | 3.22 | 4.02 | 3.89 | 4.84 | 4.8 | 3.66 | 3.9 | 2.72 | 3.13 | 4.56 | 3.76 | 0.38 |
|  | **Cys** | 0.95 | 1.04 | 1.01 | 1.15 | 1.2 | 1.06 | 1.11 | 0.82 | 1.26 | 0.9 | 0.79 | 1.15 | 0.96 | 0.72 |
|  | **Summation^**^** | 25.64 | 25.4 | 26.21 | 27.2 | 27.62 | 26.95 | 26.71 | 25.99 | 26.98 | 26.94 | 27.14 | 26.22 | 27.12 | **0** |
| **Charged amino acids** | **His** | 2.1 | 1.85 | 1.91 | 1.92 | 1.83 | 1.9 | 1.95 | 2.02 | 1.97 | 1.99 | 1.93 | 1.88 | 2.12 | 0.69 |
|  | **Lys** | 2.75 | 2.48 | 2.72 | 3.26 | 3.45 | 3.42 | 3.26 | 2.87 | 2.76 | 2.75 | 2.94 | 3.03 | 2.86 | 0.07 |
|  | **Arg** | 1.31 | 1.34 | 1.34 | 1.34 | 1.34 | 1.3 | 1.36 | 1.31 | 1.26 | 1.41 | 1.39 | 1.34 | 1.35 | 0.58 |
|  | **Asp** | 1.88 | 1.88 | 1.88 | 1.86 | 1.83 | 1.74 | 1.76 | 1.88 | 1.86 | 1.93 | 1.99 | 2.08 | 1.81 | 0.95 |
|  | **Glu** | 2.1 | 2.29 | 2.45 | 2.49 | 2.6 | 2.25 | 2.39 | 2.32 | 2.43 | 2.18 | 2.37 | 2.27 | 2.34 | 0.08 |
|  | **Summation^**^** | 10.14 | 9.84 | 10.3 | 10.86 | 11.06 | 10.62 | 10.72 | 10.4 | 10.27 | 10.26 | 10.62 | 10.6 | 10.47 | **0.01** |
| Note: **: highly significant difference between the hadal and shallow water species (*p* < 0.01). *: significant difference between the deep sea and shallow water species (0.01 < *p* < 0.05). | | | | | | | | | | | | | | | |

| **Table S5**. **Summary of codon usages of mitochondrial PCGs for amphipods from different superfamilies in the present study.** | | | | | | | | | | | | | | | | | | | | | | |
| --- | --- | --- | --- | --- | --- | --- | --- | --- | --- | --- | --- | --- | --- | --- | --- | --- | --- | --- | --- | --- | --- | --- |
|  |  | *Halice* sp. MT-2017 | | | *Hirondellea gigas* | | | *Onisimus nanseni* | | | *Caprella scaura* | | | *Caprella mutica* | | | *Metacrangonyx longipes* | | | *Longipodacrangonyx* sp | | |
| amino acid | codon | no. | % | RSCU | no. | % | RSCU | no. | % | RSCU | no. | % | RSCU | no. | % | RSCU | no. | % | RSCU | no. | % | RSCU |
| Phe | TTT | 341 | 9.26 | 1.91 | 333 | 9.04 | 1.98 | 289 | 7.85 | 1.84 | 226 | 6.16 | 1.56 | 259 | 7.06 | 1.68 | 325 | 8.80 | 1.8 | 298 | 8.06 | 1.67 |
|  | TTC | 17 | 0.46 | 0.09 | 3 | 0.08 | 0.02 | 25 | 0.68 | 0.16 | 64 | 1.74 | 0.44 | 50 | 1.36 | 0.32 | 37 | 1.00 | 0.2 | 59 | 1.60 | 0.33 |
| Leu | TTA | 366 | 9.94 | 3.89 | 294 | 7.98 | 3 | 334 | 9.07 | 3.44 | 256 | 6.98 | 2.71 | 249 | 6.79 | 2.63 | 396 | 10.72 | 4.25 | 342 | 9.25 | 3.64 |
|  | TTG | 91 | 2.47 | 0.97 | 175 | 4.75 | 1.79 | 65 | 1.76 | 0.67 | 66 | 1.80 | 0.7 | 72 | 1.96 | 0.76 | 41 | 1.11 | 0.44 | 76 | 2.06 | 0.81 |
|  | CTT | 47 | 1.28 | 0.5 | 106 | 2.88 | 1.08 | 87 | 2.36 | 0.9 | 88 | 2.40 | 0.93 | 120 | 3.27 | 1.27 | 68 | 1.84 | 0.73 | 69 | 1.87 | 0.73 |
|  | CTC | 8 | 0.22 | 0.08 | 1 | 0.03 | 0.01 | 11 | 0.30 | 0.11 | 34 | 0.93 | 0.36 | 30 | 0.82 | 0.32 | 1 | 0.03 | 0.01 | 17 | 0.46 | 0.18 |
|  | CTA | 42 | 1.14 | 0.45 | 7 | 0.19 | 0.07 | 77 | 2.09 | 0.79 | 91 | 2.48 | 0.96 | 80 | 2.18 | 0.84 | 47 | 1.27 | 0.5 | 54 | 1.46 | 0.57 |
|  | CTG | 11 | 0.30 | 0.12 | 5 | 0.14 | 0.05 | 9 | 0.24 | 0.09 | 31 | 0.85 | 0.33 | 18 | 0.49 | 0.19 | 6 | 0.16 | 0.06 | 6 | 0.16 | 0.06 |
| Ile | ATT | 263 | 7.14 | 1.76 | 265 | 7.20 | 1.96 | 246 | 6.68 | 1.72 | 190 | 5.18 | 1.41 | 189 | 5.16 | 1.31 | 339 | 9.18 | 1.85 | 307 | 8.30 | 1.67 |
|  | ATC | 36 | 0.98 | 0.24 | 6 | 0.16 | 0.04 | 40 | 1.09 | 0.28 | 79 | 2.15 | 0.59 | 99 | 2.70 | 0.69 | 27 | 0.73 | 0.15 | 61 | 1.65 | 0.33 |
| Met | ATA | 205 | 5.57 | 1.6 | 169 | 4.59 | 1.49 | 186 | 5.05 | 1.67 | 171 | 4.66 | 1.5 | 191 | 5.21 | 1.69 | 252 | 6.82 | 1.87 | 228 | 6.17 | 1.65 |
|  | ATG | 52 | 1.41 | 0.4 | 58 | 1.57 | 0.51 | 37 | 1.00 | 0.33 | 57 | 1.55 | 0.5 | 35 | 0.95 | 0.31 | 18 | 0.49 | 0.13 | 48 | 1.30 | 0.35 |
| Val | GTT | 103 | 2.80 | 1.46 | 166 | 4.51 | 2.18 | 100 | 2.72 | 1.49 | 101 | 2.75 | 1.47 | 87 | 2.37 | 1.51 | 96 | 2.60 | 1.73 | 85 | 2.30 | 1.59 |
|  | GTC | 12 | 0.33 | 0.17 | 4 | 0.11 | 0.05 | 18 | 0.49 | 0.27 | 47 | 1.28 | 0.69 | 30 | 0.82 | 0.52 | 16 | 0.43 | 0.29 | 9 | 0.24 | 0.17 |
|  | GTA | 118 | 3.20 | 1.67 | 77 | 2.09 | 1.01 | 109 | 2.96 | 1.62 | 73 | 1.99 | 1.07 | 80 | 2.18 | 1.39 | 91 | 2.46 | 1.64 | 87 | 2.35 | 1.63 |
|  | GTG | 50 | 1.36 | 0.71 | 57 | 1.55 | 0.75 | 42 | 1.14 | 0.62 | 53 | 1.44 | 0.77 | 33 | 0.90 | 0.57 | 19 | 0.51 | 0.34 | 33 | 0.89 | 0.62 |
| Ser | TCT | 111 | 3.01 | 2.35 | 172 | 4.67 | 3.21 | 113 | 3.07 | 2.18 | 113 | 3.08 | 2.41 | 129 | 3.52 | 2.54 | 128 | 3.47 | 2.65 | 120 | 3.25 | 2.53 |
|  | TCC | 9 | 0.24 | 0.19 | 2 | 0.05 | 0.04 | 10 | 0.27 | 0.19 | 38 | 1.04 | 0.81 | 45 | 1.23 | 0.89 | 13 | 0.35 | 0.27 | 20 | 0.54 | 0.42 |
|  | TCA | 49 | 1.33 | 1.04 | 34 | 0.92 | 0.64 | 71 | 1.93 | 1.37 | 58 | 1.58 | 1.24 | 62 | 1.69 | 1.22 | 55 | 1.49 | 1.14 | 60 | 1.62 | 1.26 |
|  | TCG | 18 | 0.49 | 0.38 | 12 | 0.33 | 0.22 | 9 | 0.24 | 0.17 | 12 | 0.33 | 0.26 | 22 | 0.60 | 0.43 | 10 | 0.27 | 0.21 | 8 | 0.22 | 0.17 |
|  | AGT | 63 | 1.71 | 1.33 | 94 | 2.55 | 1.76 | 62 | 1.68 | 1.2 | 31 | 0.85 | 0.66 | 39 | 1.06 | 0.77 | 42 | 1.14 | 0.87 | 45 | 1.22 | 0.95 |
|  | AGC | 8 | 0.22 | 0.17 | 3 | 0.08 | 0.06 | 19 | 0.52 | 0.37 | 21 | 0.57 | 0.45 | 15 | 0.41 | 0.3 | 7 | 0.19 | 0.15 | 8 | 0.22 | 0.17 |
|  | AGA | 74 | 2.01 | 1.57 | 66 | 1.79 | 1.23 | 101 | 2.74 | 1.95 | 62 | 1.69 | 1.32 | 64 | 1.75 | 1.26 | 103 | 2.79 | 2.13 | 91 | 2.46 | 1.92 |
|  | AGG | 46 | 1.25 | 0.97 | 45 | 1.22 | 0.84 | 30 | 0.81 | 0.58 | 40 | 1.09 | 0.85 | 30 | 0.82 | 0.59 | 28 | 0.76 | 0.58 | 28 | 0.76 | 0.59 |
| Pro | CCT | 72 | 1.95 | 2.3 | 109 | 2.96 | 3.3 | 86 | 2.34 | 2.28 | 81 | 2.21 | 2.17 | 82 | 2.24 | 2.23 | 84 | 2.27 | 2.9 | 78 | 2.11 | 2.56 |
|  | CCC | 16 | 0.43 | 0.51 | 1 | 0.03 | 0.03 | 21 | 0.57 | 0.56 | 31 | 0.85 | 0.83 | 36 | 0.98 | 0.98 | 10 | 0.27 | 0.34 | 19 | 0.51 | 0.62 |
|  | CCA | 24 | 0.65 | 0.77 | 16 | 0.43 | 0.48 | 35 | 0.95 | 0.93 | 27 | 0.74 | 0.72 | 22 | 0.60 | 0.6 | 18 | 0.49 | 0.62 | 12 | 0.32 | 0.39 |
|  | CCG | 13 | 0.35 | 0.42 | 6 | 0.16 | 0.18 | 9 | 0.24 | 0.24 | 10 | 0.27 | 0.27 | 7 | 0.19 | 0.19 | 4 | 0.11 | 0.14 | 13 | 0.35 | 0.43 |
| Thr | ACT | 89 | 2.42 | 2.16 | 93 | 2.53 | 2.72 | 90 | 2.44 | 1.85 | 91 | 2.48 | 1.6 | 109 | 2.97 | 1.95 | 91 | 2.46 | 2.39 | 76 | 2.06 | 2.07 |
|  | ACC | 11 | 0.30 | 0.27 | 1 | 0.03 | 0.03 | 26 | 0.71 | 0.53 | 53 | 1.44 | 0.93 | 34 | 0.93 | 0.61 | 16 | 0.43 | 0.42 | 16 | 0.43 | 0.44 |
|  | ACA | 47 | 1.28 | 1.14 | 35 | 0.95 | 1.02 | 71 | 1.93 | 1.46 | 66 | 1.80 | 1.16 | 71 | 1.94 | 1.27 | 42 | 1.14 | 1.11 | 47 | 1.27 | 1.28 |
|  | ACG | 18 | 0.49 | 0.44 | 8 | 0.22 | 0.23 | 8 | 0.22 | 0.16 | 17 | 0.46 | 0.3 | 10 | 0.27 | 0.18 | 3 | 0.08 | 0.08 | 8 | 0.22 | 0.22 |
| Ala | GCT | 65 | 1.76 | 1.71 | 132 | 3.58 | 3.02 | 115 | 3.12 | 2.4 | 86 | 2.34 | 1.74 | 82 | 2.24 | 1.74 | 77 | 2.08 | 2.46 | 74 | 2.00 | 2.14 |
|  | GCC | 28 | 0.76 | 0.74 | 4 | 0.11 | 0.09 | 26 | 0.71 | 0.54 | 64 | 1.74 | 1.29 | 57 | 1.55 | 1.21 | 14 | 0.38 | 0.45 | 31 | 0.84 | 0.9 |
|  | GCA | 38 | 1.03 | 1 | 30 | 0.81 | 0.69 | 43 | 1.17 | 0.9 | 29 | 0.79 | 0.59 | 35 | 0.95 | 0.74 | 29 | 0.79 | 0.93 | 21 | 0.57 | 0.61 |
|  | GCG | 21 | 0.57 | 0.55 | 9 | 0.24 | 0.21 | 8 | 0.22 | 0.17 | 19 | 0.52 | 0.38 | 14 | 0.38 | 0.3 | 5 | 0.14 | 0.16 | 12 | 0.32 | 0.35 |
| Tyr | TAT | 158 | 4.29 | 1.81 | 142 | 3.86 | 1.93 | 83 | 2.25 | 1.29 | 79 | 2.15 | 1.16 | 65 | 1.77 | 1 | 150 | 4.06 | 1.63 | 139 | 3.76 | 1.53 |
|  | TAC | 17 | 0.46 | 0.19 | 5 | 0.14 | 0.07 | 46 | 1.25 | 0.71 | 57 | 1.55 | 0.84 | 65 | 1.77 | 1 | 34 | 0.92 | 0.37 | 43 | 1.16 | 0.47 |
| His | CAT | 69 | 1.87 | 1.79 | 60 | 1.63 | 1.76 | 34 | 0.92 | 0.97 | 29 | 0.79 | 0.83 | 33 | 0.90 | 0.99 | 60 | 1.62 | 1.71 | 53 | 1.43 | 1.47 |
|  | CAC | 8 | 0.22 | 0.21 | 8 | 0.22 | 0.24 | 36 | 0.98 | 1.03 | 41 | 1.12 | 1.17 | 34 | 0.93 | 1.01 | 10 | 0.27 | 0.29 | 19 | 0.51 | 0.53 |
| Gln | CAA | 35 | 0.95 | 1.27 | 32 | 0.87 | 0.98 | 58 | 1.57 | 1.71 | 50 | 1.36 | 1.49 | 55 | 1.50 | 1.75 | 50 | 1.35 | 1.89 | 45 | 1.22 | 1.58 |
|  | CAG | 20 | 0.54 | 0.73 | 33 | 0.90 | 1.02 | 10 | 0.27 | 0.29 | 17 | 0.46 | 0.51 | 8 | 0.22 | 0.25 | 3 | 0.08 | 0.11 | 12 | 0.32 | 0.42 |
| Asn | AAT | 113 | 3.07 | 1.7 | 110 | 2.99 | 1.88 | 71 | 1.93 | 1.2 | 82 | 2.24 | 1.12 | 78 | 2.13 | 1.1 | 152 | 4.11 | 1.71 | 142 | 3.84 | 1.6 |
|  | AAC | 20 | 0.54 | 0.3 | 7 | 0.19 | 0.12 | 47 | 1.28 | 0.8 | 65 | 1.77 | 0.88 | 64 | 1.75 | 0.9 | 26 | 0.70 | 0.29 | 35 | 0.95 | 0.4 |
| Lys | AAA | 73 | 1.98 | 1.45 | 52 | 1.41 | 1.14 | 91 | 2.47 | 1.82 | 105 | 2.86 | 1.76 | 117 | 3.19 | 1.86 | 115 | 3.11 | 1.83 | 100 | 2.70 | 1.67 |
|  | AAG | 28 | 0.76 | 0.55 | 39 | 1.06 | 0.86 | 9 | 0.24 | 0.18 | 14 | 0.38 | 0.24 | 9 | 0.25 | 0.14 | 11 | 0.30 | 0.17 | 20 | 0.54 | 0.33 |
| Asp | GAT | 58 | 1.57 | 1.68 | 65 | 1.76 | 1.88 | 34 | 0.92 | 0.99 | 34 | 0.93 | 1 | 26 | 0.71 | 0.78 | 54 | 1.46 | 1.69 | 47 | 1.27 | 1.45 |
|  | GAC | 11 | 0.30 | 0.32 | 4 | 0.11 | 0.12 | 35 | 0.95 | 1.01 | 34 | 0.93 | 1 | 41 | 1.12 | 1.22 | 10 | 0.27 | 0.31 | 18 | 0.49 | 0.55 |
| Glu | GAA | 57 | 1.55 | 1.48 | 35 | 0.95 | 0.83 | 56 | 1.52 | 1.24 | 59 | 1.61 | 1.3 | 59 | 1.61 | 1.24 | 57 | 1.54 | 1.37 | 58 | 1.57 | 1.32 |
|  | GAG | 20 | 0.54 | 0.52 | 49 | 1.33 | 1.17 | 34 | 0.92 | 0.76 | 32 | 0.87 | 0.7 | 36 | 0.98 | 0.76 | 26 | 0.70 | 0.63 | 30 | 0.81 | 0.68 |
| Cys | TGT | 33 | 0.90 | 1.89 | 37 | 1.00 | 1.95 | 29 | 0.79 | 1.57 | 27 | 0.74 | 1.29 | 32 | 0.87 | 1.45 | 37 | 1.00 | 1.9 | 31 | 0.84 | 1.51 |
|  | TGC | 2 | 0.05 | 0.11 | 1 | 0.03 | 0.05 | 8 | 0.22 | 0.43 | 15 | 0.41 | 0.71 | 12 | 0.33 | 0.55 | 2 | 0.05 | 0.1 | 10 | 0.27 | 0.49 |
| Trp | TGA | 60 | 1.63 | 1.25 | 43 | 1.17 | 0.91 | 66 | 1.79 | 1.5 | 36 | 0.98 | 0.86 | 45 | 1.23 | 1.03 | 61 | 1.65 | 1.45 | 58 | 1.57 | 1.4 |
|  | TGG | 36 | 0.98 | 0.75 | 51 | 1.38 | 1.09 | 22 | 0.60 | 0.5 | 48 | 1.31 | 1.14 | 42 | 1.15 | 0.97 | 23 | 0.62 | 0.55 | 25 | 0.68 | 0.6 |
| Arg | CGT | 23 | 0.62 | 1.92 | 26 | 0.71 | 2.12 | 16 | 0.43 | 1.31 | 6 | 0.16 | 0.49 | 10 | 0.27 | 0.82 | 22 | 0.60 | 1.83 | 20 | 0.54 | 1.6 |
|  | CGC | 2 | 0.05 | 0.17 | 0 | 0.00 | 0 | 3 | 0.08 | 0.24 | 5 | 0.14 | 0.41 | 7 | 0.19 | 0.57 | 1 | 0.03 | 0.08 | 3 | 0.08 | 0.24 |
|  | CGA | 15 | 0.41 | 1.25 | 7 | 0.19 | 0.57 | 22 | 0.60 | 1.8 | 21 | 0.57 | 1.71 | 19 | 0.52 | 1.55 | 21 | 0.57 | 1.75 | 24 | 0.65 | 1.92 |
|  | CGG | 8 | 0.22 | 0.67 | 16 | 0.43 | 1.31 | 8 | 0.22 | 0.65 | 17 | 0.46 | 1.39 | 13 | 0.35 | 1.06 | 4 | 0.11 | 0.33 | 3 | 0.08 | 0.24 |
| Gly | GGT | 59 | 1.60 | 1.06 | 162 | 4.40 | 2.59 | 96 | 2.61 | 1.71 | 64 | 1.74 | 1.24 | 68 | 1.85 | 1.39 | 56 | 1.52 | 1.15 | 73 | 1.97 | 1.6 |
|  | GGC | 14 | 0.38 | 0.25 | 11 | 0.30 | 0.18 | 34 | 0.92 | 0.61 | 28 | 0.76 | 0.54 | 22 | 0.60 | 0.45 | 21 | 0.57 | 0.43 | 18 | 0.49 | 0.39 |
|  | GGA | 84 | 2.28 | 1.51 | 18 | 0.49 | 0.29 | 45 | 1.22 | 0.8 | 38 | 1.04 | 0.74 | 47 | 1.28 | 0.96 | 75 | 2.03 | 1.55 | 45 | 1.22 | 0.98 |
|  | GGG | 65 | 1.76 | 1.17 | 59 | 1.60 | 0.94 | 49 | 1.33 | 0.88 | 76 | 2.07 | 1.48 | 59 | 1.61 | 1.2 | 42 | 1.14 | 0.87 | 47 | 1.27 | 1.03 |
| Stop | TAA | 11 | 0.30 | 1.69 | 10 | 0.27 | 1.54 | 11 | 0.30 | 1.69 | 12 | 0.33 | 1.85 | 13 | 0.35 | 2 | 12 | 0.32 | 1.85 | 12 | 0.32 | 1.85 |
|  | TAG | 2 | 0.05 | 0.31 | 3 | 0.08 | 0.46 | 2 | 0.05 | 0.31 | 1 | 0.03 | 0.15 | 0 | 0.00 | 0 | 1 | 0.03 | 0.15 | 1 | 0.03 | 0.15 |
|  |  | *Pseudoniphargus gorbeanus* | | | *Pseudoniphargus sorbasiensis* | | | *Brachyuropus grewingkii* | | | *Eulimnogammarus vittatus* | | | *Parhyale hawaiiensis* | | | *Gondogeneia antarctica* | | |  |  |  |
| amino acid | codon | no. | % | RSCU | no. | % | RSCU | no. | % | RSCU | no. | % | RSCU | no. | % | RSCU | no. | % | RSCU |  |  |  |
| Phe | TTT | 266 | 7.24 | 1.62 | 268 | 7.29 | 1.61 | 218 | 5.91 | 1.6 | 245 | 6.65 | 1.68 | 304 | 8.27 | 1.79 | 236 | 6.46 | 1.58 |  |  |  |
|  | TTC | 63 | 1.71 | 0.38 | 65 | 1.77 | 0.39 | 55 | 1.49 | 0.4 | 46 | 1.25 | 0.32 | 36 | 0.98 | 0.21 | 63 | 1.73 | 0.42 |  |  |  |
| Leu | TTA | 271 | 7.37 | 2.88 | 274 | 7.46 | 3.06 | 216 | 5.86 | 2.19 | 276 | 7.49 | 2.94 | 373 | 10.15 | 4.18 | 294 | 8.05 | 3.07 |  |  |  |
|  | TTG | 88 | 2.39 | 0.94 | 78 | 2.12 | 0.87 | 68 | 1.84 | 0.69 | 57 | 1.55 | 0.61 | 38 | 1.03 | 0.43 | 66 | 1.81 | 0.69 |  |  |  |
|  | CTT | 69 | 1.88 | 0.73 | 62 | 1.69 | 0.69 | 90 | 2.44 | 0.91 | 86 | 2.33 | 0.92 | 53 | 1.44 | 0.59 | 94 | 2.57 | 0.98 |  |  |  |
|  | CTC | 38 | 1.03 | 0.4 | 10 | 0.27 | 0.11 | 39 | 1.06 | 0.39 | 9 | 0.24 | 0.1 | 8 | 0.22 | 0.09 | 25 | 0.68 | 0.26 |  |  |  |
|  | CTA | 82 | 2.23 | 0.87 | 94 | 2.56 | 1.05 | 134 | 3.63 | 1.36 | 118 | 3.20 | 1.26 | 59 | 1.61 | 0.66 | 77 | 2.11 | 0.8 |  |  |  |
|  | CTG | 16 | 0.44 | 0.17 | 20 | 0.54 | 0.22 | 46 | 1.25 | 0.47 | 17 | 0.46 | 0.18 | 4 | 0.11 | 0.04 | 19 | 0.52 | 0.2 |  |  |  |
| Ile | ATT | 252 | 6.86 | 1.51 | 257 | 6.99 | 1.6 | 156 | 4.23 | 1.44 | 188 | 5.10 | 1.44 | 315 | 8.57 | 1.72 | 224 | 6.13 | 1.52 |  |  |  |
|  | ATC | 81 | 2.20 | 0.49 | 64 | 1.74 | 0.4 | 60 | 1.63 | 0.56 | 73 | 1.98 | 0.56 | 51 | 1.39 | 0.28 | 70 | 1.92 | 0.48 |  |  |  |
| Met | ATA | 214 | 5.82 | 1.69 | 236 | 6.42 | 1.61 | 148 | 4.01 | 1.62 | 187 | 5.07 | 1.56 | 237 | 6.45 | 1.76 | 177 | 4.85 | 1.6 |  |  |  |
|  | ATG | 40 | 1.09 | 0.31 | 58 | 1.58 | 0.39 | 35 | 0.95 | 0.38 | 52 | 1.41 | 0.44 | 33 | 0.90 | 0.24 | 44 | 1.20 | 0.4 |  |  |  |
| Val | GTT | 90 | 2.45 | 1.51 | 111 | 3.02 | 1.75 | 100 | 2.71 | 1.28 | 114 | 3.09 | 1.62 | 94 | 2.56 | 1.73 | 103 | 2.82 | 1.6 |  |  |  |
|  | GTC | 19 | 0.52 | 0.32 | 9 | 0.24 | 0.14 | 58 | 1.57 | 0.74 | 25 | 0.68 | 0.36 | 13 | 0.35 | 0.24 | 20 | 0.55 | 0.31 |  |  |  |
|  | GTA | 90 | 2.45 | 1.51 | 95 | 2.59 | 1.5 | 96 | 2.60 | 1.23 | 90 | 2.44 | 1.28 | 85 | 2.31 | 1.57 | 83 | 2.27 | 1.29 |  |  |  |
|  | GTG | 39 | 1.06 | 0.66 | 39 | 1.06 | 0.61 | 58 | 1.57 | 0.74 | 52 | 1.41 | 0.74 | 25 | 0.68 | 0.46 | 51 | 1.40 | 0.79 |  |  |  |
| Ser | TCT | 103 | 2.80 | 1.95 | 102 | 2.78 | 2 | 78 | 2.11 | 1.51 | 99 | 2.69 | 1.96 | 121 | 3.29 | 2.6 | 94 | 2.57 | 1.79 |  |  |  |
|  | TCC | 46 | 1.25 | 0.87 | 23 | 0.63 | 0.45 | 46 | 1.25 | 0.89 | 30 | 0.81 | 0.59 | 9 | 0.24 | 0.19 | 35 | 0.96 | 0.67 |  |  |  |
|  | TCA | 74 | 2.01 | 1.4 | 101 | 2.75 | 1.99 | 47 | 1.27 | 0.91 | 59 | 1.60 | 1.17 | 64 | 1.74 | 1.37 | 89 | 2.44 | 1.69 |  |  |  |
|  | TCG | 15 | 0.41 | 0.28 | 9 | 0.24 | 0.18 | 21 | 0.57 | 0.41 | 12 | 0.33 | 0.24 | 7 | 0.19 | 0.15 | 9 | 0.25 | 0.17 |  |  |  |
|  | AGT | 41 | 1.12 | 0.78 | 52 | 1.41 | 1.02 | 38 | 1.03 | 0.74 | 55 | 1.49 | 1.09 | 55 | 1.50 | 1.18 | 58 | 1.59 | 1.1 |  |  |  |
|  | AGC | 15 | 0.41 | 0.28 | 7 | 0.19 | 0.14 | 27 | 0.73 | 0.52 | 21 | 0.57 | 0.42 | 9 | 0.24 | 0.19 | 15 | 0.41 | 0.29 |  |  |  |
|  | AGA | 82 | 2.23 | 1.55 | 78 | 2.12 | 1.53 | 80 | 2.17 | 1.55 | 89 | 2.41 | 1.76 | 87 | 2.37 | 1.87 | 85 | 2.33 | 1.62 |  |  |  |
|  | AGG | 47 | 1.28 | 0.89 | 35 | 0.95 | 0.69 | 76 | 2.06 | 1.47 | 39 | 1.06 | 0.77 | 21 | 0.57 | 0.45 | 36 | 0.99 | 0.68 |  |  |  |
| Pro | CCT | 76 | 2.07 | 2.27 | 60 | 1.63 | 1.88 | 85 | 2.30 | 2.28 | 72 | 1.95 | 2.07 | 82 | 2.23 | 2.47 | 88 | 2.41 | 2.46 |  |  |  |
|  | CCC | 23 | 0.63 | 0.69 | 14 | 0.38 | 0.44 | 36 | 0.98 | 0.97 | 23 | 0.62 | 0.66 | 10 | 0.27 | 0.3 | 22 | 0.60 | 0.62 |  |  |  |
|  | CCA | 29 | 0.79 | 0.87 | 48 | 1.31 | 1.5 | 23 | 0.62 | 0.62 | 30 | 0.81 | 0.86 | 36 | 0.98 | 1.08 | 29 | 0.79 | 0.81 |  |  |  |
|  | CCG | 6 | 0.16 | 0.18 | 6 | 0.16 | 0.19 | 5 | 0.14 | 0.13 | 14 | 0.38 | 0.4 | 5 | 0.14 | 0.15 | 4 | 0.11 | 0.11 |  |  |  |
| Thr | ACT | 64 | 1.74 | 1.63 | 63 | 1.71 | 1.46 | 88 | 2.39 | 1.55 | 107 | 2.90 | 1.87 | 79 | 2.15 | 1.99 | 55 | 1.51 | 1.16 |  |  |  |
|  | ACC | 33 | 0.90 | 0.84 | 23 | 0.63 | 0.53 | 65 | 1.76 | 1.15 | 39 | 1.06 | 0.68 | 14 | 0.38 | 0.35 | 34 | 0.93 | 0.72 |  |  |  |
|  | ACA | 52 | 1.41 | 1.32 | 84 | 2.29 | 1.94 | 66 | 1.79 | 1.16 | 68 | 1.84 | 1.19 | 58 | 1.58 | 1.46 | 91 | 2.49 | 1.93 |  |  |  |
|  | ACG | 8 | 0.22 | 0.2 | 3 | 0.08 | 0.07 | 8 | 0.22 | 0.14 | 15 | 0.41 | 0.26 | 8 | 0.22 | 0.2 | 9 | 0.25 | 0.19 |  |  |  |
| Ala | GCT | 66 | 1.80 | 1.59 | 70 | 1.90 | 1.89 | 99 | 2.68 | 1.6 | 98 | 2.66 | 1.95 | 112 | 3.05 | 2.6 | 79 | 2.16 | 1.82 |  |  |  |
|  | GCC | 54 | 1.47 | 1.3 | 28 | 0.76 | 0.76 | 70 | 1.90 | 1.13 | 47 | 1.28 | 0.94 | 12 | 0.33 | 0.28 | 41 | 1.12 | 0.94 |  |  |  |
|  | GCA | 33 | 0.90 | 0.8 | 38 | 1.03 | 1.03 | 53 | 1.44 | 0.85 | 42 | 1.14 | 0.84 | 36 | 0.98 | 0.84 | 43 | 1.18 | 0.99 |  |  |  |
|  | GCG | 13 | 0.35 | 0.31 | 12 | 0.33 | 0.32 | 26 | 0.70 | 0.42 | 14 | 0.38 | 0.28 | 12 | 0.33 | 0.28 | 11 | 0.30 | 0.25 |  |  |  |
| Tyr | TAT | 102 | 2.77 | 1.38 | 115 | 3.13 | 1.47 | 69 | 1.87 | 0.94 | 88 | 2.39 | 1.16 | 116 | 3.16 | 1.44 | 92 | 2.52 | 1.26 |  |  |  |
|  | TAC | 46 | 1.25 | 0.62 | 41 | 1.12 | 0.53 | 78 | 2.11 | 1.06 | 64 | 1.74 | 0.84 | 45 | 1.22 | 0.56 | 54 | 1.48 | 0.74 |  |  |  |
| His | CAT | 39 | 1.06 | 1.05 | 39 | 1.06 | 1.08 | 37 | 1.00 | 1.01 | 29 | 0.79 | 0.82 | 51 | 1.39 | 1.48 | 39 | 1.07 | 1.01 |  |  |  |
|  | CAC | 35 | 0.95 | 0.95 | 33 | 0.90 | 0.92 | 36 | 0.98 | 0.99 | 42 | 1.14 | 1.18 | 18 | 0.49 | 0.52 | 38 | 1.04 | 0.99 |  |  |  |
| Gln | CAA | 46 | 1.25 | 1.53 | 50 | 1.36 | 1.59 | 41 | 1.11 | 1.17 | 51 | 1.38 | 1.5 | 48 | 1.31 | 1.66 | 46 | 1.26 | 1.56 |  |  |  |
|  | CAG | 14 | 0.38 | 0.47 | 13 | 0.35 | 0.41 | 29 | 0.79 | 0.83 | 17 | 0.46 | 0.5 | 10 | 0.27 | 0.34 | 13 | 0.36 | 0.44 |  |  |  |
| Asn | AAT | 95 | 2.58 | 1.42 | 110 | 2.99 | 1.54 | 43 | 1.17 | 0.86 | 63 | 1.71 | 1.1 | 140 | 3.81 | 1.68 | 76 | 2.08 | 1.11 |  |  |  |
|  | AAC | 39 | 1.06 | 0.58 | 33 | 0.90 | 0.46 | 57 | 1.55 | 1.14 | 52 | 1.41 | 0.9 | 27 | 0.73 | 0.32 | 61 | 1.67 | 0.89 |  |  |  |
| Lys | AAA | 78 | 2.12 | 1.49 | 83 | 2.26 | 1.64 | 66 | 1.79 | 1.31 | 91 | 2.47 | 1.69 | 99 | 2.69 | 1.78 | 86 | 2.35 | 1.65 |  |  |  |
|  | AAG | 27 | 0.73 | 0.51 | 18 | 0.49 | 0.36 | 35 | 0.95 | 0.69 | 17 | 0.46 | 0.31 | 12 | 0.33 | 0.22 | 18 | 0.49 | 0.35 |  |  |  |
| Asp | GAT | 47 | 1.28 | 1.36 | 43 | 1.17 | 1.26 | 26 | 0.70 | 0.73 | 36 | 0.98 | 0.99 | 55 | 1.50 | 1.45 | 50 | 1.37 | 1.52 |  |  |  |
|  | GAC | 22 | 0.60 | 0.64 | 25 | 0.68 | 0.74 | 45 | 1.22 | 1.27 | 37 | 1.00 | 1.01 | 21 | 0.57 | 0.55 | 16 | 0.44 | 0.48 |  |  |  |
| Glu | GAA | 51 | 1.39 | 1.2 | 51 | 1.39 | 1.15 | 36 | 0.98 | 0.9 | 43 | 1.17 | 0.99 | 54 | 1.47 | 1.3 | 51 | 1.40 | 1.2 |  |  |  |
|  | GAG | 34 | 0.92 | 0.8 | 38 | 1.03 | 0.85 | 44 | 1.19 | 1.1 | 44 | 1.19 | 1.01 | 29 | 0.79 | 0.7 | 34 | 0.93 | 0.8 |  |  |  |
| Cys | TGT | 25 | 0.68 | 1.67 | 37 | 1.01 | 1.61 | 25 | 0.68 | 1.52 | 21 | 0.57 | 1.45 | 37 | 1.01 | 1.76 | 26 | 0.71 | 1.49 |  |  |  |
|  | TGC | 5 | 0.14 | 0.33 | 9 | 0.24 | 0.39 | 8 | 0.22 | 0.48 | 8 | 0.22 | 0.55 | 5 | 0.14 | 0.24 | 9 | 0.25 | 0.51 |  |  |  |
| Trp | TGA | 70 | 1.90 | 1.49 | 66 | 1.80 | 1.5 | 55 | 1.49 | 1.18 | 55 | 1.49 | 1.22 | 72 | 1.96 | 1.64 | 54 | 1.48 | 1.32 |  |  |  |
|  | TGG | 24 | 0.65 | 0.51 | 22 | 0.60 | 0.5 | 38 | 1.03 | 0.82 | 35 | 0.95 | 0.78 | 16 | 0.44 | 0.36 | 28 | 0.77 | 0.68 |  |  |  |
| Arg | CGT | 12 | 0.33 | 1 | 16 | 0.44 | 1.39 | 12 | 0.33 | 0.92 | 17 | 0.46 | 1.33 | 23 | 0.63 | 1.88 | 17 | 0.47 | 1.39 |  |  |  |
|  | CGC | 9 | 0.24 | 0.75 | 7 | 0.19 | 0.61 | 11 | 0.30 | 0.85 | 5 | 0.14 | 0.39 | 3 | 0.08 | 0.24 | 3 | 0.08 | 0.24 |  |  |  |
|  | CGA | 16 | 0.44 | 1.33 | 18 | 0.49 | 1.57 | 15 | 0.41 | 1.15 | 19 | 0.52 | 1.49 | 18 | 0.49 | 1.47 | 15 | 0.41 | 1.22 |  |  |  |
|  | CGG | 11 | 0.30 | 0.92 | 5 | 0.14 | 0.43 | 14 | 0.38 | 1.08 | 10 | 0.27 | 0.78 | 5 | 0.14 | 0.41 | 14 | 0.38 | 1.14 |  |  |  |
| Gly | GGT | 63 | 1.71 | 1.16 | 60 | 1.63 | 1.24 | 54 | 1.46 | 0.9 | 87 | 2.36 | 1.57 | 84 | 2.29 | 1.74 | 109 | 2.98 | 1.93 |  |  |  |
|  | GGC | 21 | 0.57 | 0.39 | 30 | 0.82 | 0.62 | 49 | 1.33 | 0.81 | 26 | 0.71 | 0.47 | 20 | 0.54 | 0.41 | 33 | 0.90 | 0.58 |  |  |  |
|  | GGA | 53 | 1.44 | 0.97 | 60 | 1.63 | 1.24 | 46 | 1.25 | 0.76 | 36 | 0.98 | 0.65 | 44 | 1.20 | 0.91 | 38 | 1.04 | 0.67 |  |  |  |
|  | GGG | 81 | 2.20 | 1.49 | 44 | 1.20 | 0.91 | 92 | 2.49 | 1.53 | 72 | 1.95 | 1.3 | 45 | 1.22 | 0.93 | 46 | 1.26 | 0.81 |  |  |  |
| Stop | TAA | 11 | 0.30 | 1.69 | 12 | 0.33 | 1.85 | 10 | 0.27 | 1.54 | 11 | 0.30 | 1.69 | 11 | 0.30 | 1.69 | 12 | 0.33 | 1.85 |  |  |  |
|  | TAG | 2 | 0.05 | 0.31 | 1 | 0.03 | 0.15 | 3 | 0.08 | 0.46 | 2 | 0.05 | 0.31 | 2 | 0.05 | 0.31 | 1 | 0.03 | 0.15 |  |  |  |
| Note: “no.” denotes the number of certain codon in related species, “%” indicates the percentage of certain codon in total mitochondrial coding sequence of related species, and the “RSCU” is the relative synonymous codon usage, measured for the codon usage bias in certain amino acid. | | | | | | | | | | | | | | | | | | | |  |  |  |

**Table S6 The dN/dS ratios of the 13 PCGs for 9 amphipods.**

| Species | *atp6* | *atp8* | *cob* | *cox1* | *cox2* | *cox3* | *nad1* | *nad2* | *nad3* | *nad4* | *nad4l* | *nad5* | *nad6* |
| --- | --- | --- | --- | --- | --- | --- | --- | --- | --- | --- | --- | --- | --- |
| *Halice* sp. MT-2017 | 0.0095 | 0.8838 | 0.0564 | 0.0247 | 0.0020 | 0.0453 | 0.0304 | 0.0153 | 0.0352 | 0.0093 | 0.0943 | 0.0587 | 0.0026 |
| *Hirondellea gigas* | 0.0132 | 0.1118 | 0.0210 | 408.1 | 0.0174 | 0.0324 | 0.0350 | 0.0366 | 0.1986 | 0.0175 | 0.0029 | 0.0259 | 0.0913 |
| *Parhyale hawaiiensis* | 0.0332 | 1.0139 | 0.0182 | 0.0081 | 0.0236 | 0.0178 | 0.0294 | 0.0221 | 0.0406 | 0.0522 | 0.0497 | 0.0447 | 0.0055 |
| *Metacrangonyx longipes* | 0.0025 | 0.9223 | 0.0340 | 0.0145 | 0.0078 | 0.0451 | 0.0431 | 0.0345 | 0.0024 | 0.0789 | 0.1010 | 0.0335 | 0.0264 |
| *Gondogeneia antarctica* | 0.0220 | 0.0509 | 0.0254 | 0.0229 | 0.0720 | 0.0488 | 0.0343 | 0.0026 | 0.0342 | 0.0286 | 0.0043 | 0.0628 | 0.2745 |
| *Caprella scaura* | 0.1126 | 0.0275 | 0.0407 | 0.0446 | 0.0599 | 0.0967 | 0.0681 | 0.1304 | 0.1135 | 0.0932 | 0.0794 | 0.1105 | 0.0040 |
| *Pseudoniphargus gorbeanus* | 0.0394 | 0.0110 | 0.0182 | 0.0153 | 0.0465 | 0.0401 | 0.0819 | 0.0302 | 0.0270 | 0.0530 | 0.0022 | 0.0171 | 0.0023 |
| *Onisimus nanseni* | 0.0230 | 0.0293 | 0.0582 | 0.0212 | 0.0562 | 0.1286 | 0.0259 | 0.0633 | 0.0180 | 0.0357 | 0.0420 | 0.0584 | 0.0099 |
| *Brachyuropus grewingkii* | 0.0017 | 0.0018 | 0.0295 | 0.0372 | 0.0569 | 0.0559 | 0.0295 | 0.0946 | 0.0026 | 0.0343 | 0.0613 | 0.0674 | 0.0922 |

Note: Red font indicates that the genes with relatively smaller dN/dS ratio than the other species.

| **Table S7. Primers used to verify the accuracy of the assembled mitochondrial genome sequence.** | | | |
| --- | --- | --- | --- |
| **Name** | **Primer sequences (5’–3’)** | **Product length (bp)** | **Tm (℃)** |
| **Contig1F** | GTG GAA AGT GGA GTT GGG ACA GGA TG | 8,386 | 55 |
| **Contig1R** | CTT ATC TCA AAG AGA GTG ACG GGC AAT |  |  |
| **Contig2F** | GAA CGG GAA TGA ATG GTT TGA CAA T | 5,491 | 55 |
| **Contig2R** | CAA ATC CAC AGA AGC CCC CCT ATG |  |  |

| **Table S8. Accession no. for the sequences used in the phylogenetic analysis.** | | | | | |
| --- | --- | --- | --- | --- | --- |
| **Order** | **Superfamily** | **Family** | **Genera** | **Species** | **Genbank No.** |
| **For *cox1* barcode analysis** | | | | | |
| Amphipoda | \ | Pardaliscidae | Halice | *Halice* sp. MT-2017 | MH294484 |
| Amphipoda | \ | Pardaliscidae | Halice | *Halice* sp. 1 | MG264826 |
| Amphipoda | \ | Pardaliscidae | Halice | *Halice* sp. 2 | MG264824 |
| Amphipoda | \ | Pardaliscidae | Halice | *Halice* sp. 3 | MG264879 |
| Amphipoda | \ | Pardaliscidae | Halice | *Halice* sp. 3 | MG264809 |
| Amphipoda | \ | Pardaliscidae | Halice | Pardaliscella sp. | MG264755 |
| Amphipoda | \ | Pardaliscidae | Halice | Pardaliscella sp. | MG264796 |
| Amphipoda | \ | Pardaliscidae | Halice | *Nicippe recticaudata* | LC214958 |
| Amphipoda | \ | Pardaliscidae | Halice | *Nicippe recticaudata* | LC214960 |
| Amphipoda | Lysianassoidea | Lysianassidae | Paralicella | *Paralicella tenuipes* | KP713926 |
| Amphipoda | Lysianassoidea | Lysianassidae | Paralicella | *Paralicella tenuipes* | KP713920 |
| Amphipoda | Lysianassoidea | Lysianassidae | Hirondellea | *Hirondellea gigas* | KX497024 |
| Amphipoda | Lysianassoidea | Lysianassidae | Hirondellea | *Hirondellea gigas* | KX497025 |
| Amphipoda | Lysianassoidea | Lysianassidae | Eurythenes | *Eurythenes gryllus* | KX078264 |
| Amphipoda | Lysianassoidea | Lysianassidae | Eurythenes | *Eurythenes gryllus* | KX078265 |
| Amphipoda | \ | Alicellidae | Alicella | *Alicella gigantea* | JX436324 |
| Amphipoda | \ | Alicellidae | Alicella | *Alicella gigantea* | MF598988 |
| **For 13 concatenated mitochondrial PCGs analysis** | | | | | |
| Amphipoda | \ | Pardaliscidae | Halice | *Halice* sp. MT-2017 | MH294484 |
| Amphipoda | Hadzioidea | Metacrangonyctidae | Metacrangonyx | *Metacrangonyx longipes* | AM944817 |
| Amphipoda | Hadzioidea | Metacrangonyctidae | Longipodacrangonyx | *Longipodacrangonyx* sp. | HE860496 |
| Amphipoda | Caprelloidea | Caprellidae | Caprella | *Caprella scaura* | AB539699 |
| Amphipoda | Caprelloidea | Caprellidae | Caprella | *Caprella mutica* | GU130250 |
| Amphipoda | Allocrangonyctoidea | Allocrangonyctidae | Pseudoniphargus | *Pseudoniphargus gorbeanus* | LN871176 |
| Amphipoda | Allocrangonyctoidea | Allocrangonyctidae | Pseudoniphargus | *Pseudoniphargus sorbasiensis* | LN871175 |
| Amphipoda | Talitroidea | Hyalidae | Parhyale | *Parhyale hawaiiensis* | AY639937 & FM957526 |
| Amphipoda | Lysianassoidea | Lysianassidae | Hirondellea | *Hirondellea gigas* | KU558991 & KU558990 |
| Amphipoda | Lysianassoidea | Lysianassidae | Onisimus | *Onisimus nanseni* | FJ555185 |
| Amphipoda | Calliopioidea | Pontogeneiidae | Gondogeneia | *Gondogeneia antarctica* | JN827386 |
| Amphipoda | Gammaroidea | Acanthogammaridae | Brachyuropus | *Brachyuropus grewingkii* | KP161875 |
| Amphipoda | Gammaroidea | Eulimnogammaridae | Eulimnogammarus | *Eulimnogammarus vittatus* | KM287572 |
| Isopoda | \ | Limnoriidae | Limnoria | *Limnoria quadripunctata* | KF704000 |
| Isopoda | \ | Cirolanidae | Bathynomus | *Bathynomus* sp. | KU057374 |
| Isopoda | \ | Trachelipodidae | Trachelipus | *Trachelipus rathkii* | KR013001 |
| Isopoda | \ | Limnoriidae | Ligia | *Ligia oceanica* | DQ442914 |
